# Routine evaluation of HBV-specific T cell reactivity in chronic hepatitis B using a broad-spectrum T-cell epitope peptide library and ELISpot assay

**DOI:** 10.1186/s12967-024-05062-5

**Published:** 2024-03-11

**Authors:** Yandan Wu, Xiaotao Liu, Yuan Mao, Ruixue Ji, Lingzhi Xia, Zining Zhou, Yan Ding, Pinqing Li, Yu Zhao, Min Peng, Jie Qiu, Chuanlai Shen

**Affiliations:** 1 Department of Microbiology and Immunology, Medical School of Southeast University, Nanjing, 210009 Jiangsu China; 2 Nanjing KingMed Clinical Laboratory, Nanjing, 211899 Jiangsu China; 3 Division of Hepatitis, Nanjing Second Hospital, Nanjing Hospital affiliated to Nanjing University of Chinese Medicine, Nanjing, 210003 Jiangsu China

**Keywords:** Chronic hepatitis B, Antigen-specific T cell, T-cell epitopes, ELISpot

## Abstract

**Background:**

The clinical routine test of HBV-specific T cell reactivity is still limited due to the high polymorphisms of human leukocyte antigens (HLA) in patient cohort and the lack of universal detection kit, thus the clinical implication remains disputed.

**Methods:**

A broad-spectrum peptide library, which consists of 103 functionally validated CD8^+^ T-cell epitopes spanning overall HBsAg, HBeAg, HBx and HBpol proteins and fits to the HLA polymorphisms of Chinese and Northeast Asian populations, was grouped into eight peptide pools and was used to establish an ELISpot assay for enumerating the reactive HBV-specific T cells in PBMCs. Totally 294 HBV-infected patients including 203 ones with chronic hepatitis B (CHB), 13 ones in acute resolved stage (R), 52 ones with liver cirrhosis (LC) and 26 ones with hepatocellular carcinoma (HCC) were detected, and 33 CHB patients were longitudinally monitored for 3 times with an interval of 3–5 months.

**Results:**

The numbers of reactive HBV-specific T cells were significantly correlated with ALT level, HBsAg level, and disease stage (R, CHB, LC and HCC), and R patients displayed the strongest HBV-specific T cell reactivity while CHB patients showed the weakest one. For 203 CHB patients, the numbers of reactive HBV-specific T cells presented a significantly declined trend when the serum viral DNA load, HBsAg, HBeAg or ALT level gradually increased, but only a very low negative correlation coefficient was defined (r = − 0.21, − 0.21, − 0.27, − 0.079, respectively). Different Nucleotide Analogs (NUCs) did not bring difference on HBV-specific T cell reactivity in the same duration of treatment. NUCs/pegIFN-α combination led to much more reactive HBV-specific T cells than NUCs monotherapy. The dynamic numbers of reactive HBV-specific T cells were obviously increasing in most CHB patients undergoing routine treatment, and the longitudinal trend possess a high predictive power for the hepatitis progression 6 or 12 months later.

**Conclusion:**

The presented method could be developed into an efficient reference method for the clinical evaluation of cellular immunity. The CHB patients presenting low reactivity of HBV-specific T cells have a worse prognosis for hepatitis progression and should be treated using pegIFN-α to improve host T-cell immunity.

**Supplementary Information:**

The online version contains supplementary material available at 10.1186/s12967-024-05062-5.

## Background

Numerous researches have confirmed that T cells specific for hepatitis B virus (HBV) significantly influence the outcome of HBV infection [1–3], antiviral efficacy [4] and disease recurrence after therapy discontinuation [5–7]. However, the clinical routine evaluation of HBV-specific T cell reactivity is hampered markedly by the lack of validated T-cell epitopes covering the broad patients with distinct human leukocyte antigen (HLA) allotypes. As we recently reviewed, only 205 CD8^+^ T-cell epitopes and 79 CD4^+^ T-cell epitopes have been defined from HBV proteome by cellular functional experiments during the past 33 years, and most are restricted to several common HLA allotypes, such as HLA-A0201, A2402, B0702, DR04, and DR12 molecules [8]. Therefore, the currently defined T-cell epitope repertoire cannot cover the major populations in an indicated geographic region due to the HLA polymorphisms. As a result, the previous data about HBV-specific T cell detections were mostly from the researches using overlapping peptides (OLPs) [6, 9–13] or in silico predicted T-cell epitope peptides (PEPs) [14, 15] in ELISpot, FluroSpot or intracellular staining (ICS) assays. A small part of data was from the selected patients who carrying indicated HLA alleles by using the functionally validated T-cell epitope peptides presented by the indicated HLA allotypes [12, 16, 17]. However, the immunogenicity of OLPs and PEPs lack supports of sufficient evidences from biological validation experiments. Recent clinical experiments have further indicated that most of them should be false T-cell epitopes in real-world disease. Of the 1925 OLPs (15aa/peptide) spanning SARS-CoV-2 genome, only 280 OLPs (14.55%) could detect positive CD4^+^ T cell responses in 99 patients with SARS-CoV-2 infection [18], while 11 of 82 OLPs (15aa/peptide) covering the N protein of SARS-CoV-2 showed positive T cell reactions in 7 infected patients [19]. Similarly, of the 5600 CD8^+^ T-cell PEPs from SARS-CoV-2 proteome, only 523 peptides could detect positive CD8^+^ T cell responses in 99 patients with SARS-CoV-2 infection [18], while 29 of 499 PEPs from hepatocellular carcinoma (HCC)-associated antigens presented positive T cell responses in 46 HCC patients [20]. Therefore, using OLPs or PEPs pools to detect HBV-specific T cell responses for broad patients is a reluctant method with no other methods available, thus contradictory results and clinical implications of HBV-specific T cell responses were always reported. Therefore, it is extremely essential to establish the broad-spectrum peptide library, which consists of functionally validated T-cell epitopes of HBV proteome and fits to the herd HLA polymorphisms of indicated regional populations, for the routine detection of real-world HBV-specific T cell responses for random patients. In addition, most clinical researches focus on HBV-specific CD4^+^ T cell detection and cross-sectional evaluation [6, 9–13], the information about HBV-specific CD8^+^ T cells and longitudinal trend is also limited.

In our recent works, HBV CD8^+^ T-cell epitopes presented by thirteen prevalent HLA-A allotypes which gather a total gene frequency of around 95% in Chinese and Northeast Asian populations have been defined from overall HBsAg, HBeAg, HBx and HBpol proteins and validated by multiple experiments [21]. In this study, 103 validated epitope peptides were finally used to establish the universal ELISpot assay and evaluate HBV-specific T cell reactivity by cross-sectional and longitudinal routine detection for Chinese patients and followed by the correlation analyses with clinical features, anti-viral treatments and sero-virological parameters.

## Methods

### Patient cohort and samples

A total of 294 HBV-infected patients were enrolled in this study from the Division of Hepatitis at Nanjing Second Hospital. According to the EASL 2017 Clinical Practice Guidelines on the management of hepatitis B virus infection [7], 203 patients had clinical, biochemical and virological evidences of chronic hepatitis B (CHB) with HBsAg positiveness at least 6 months. According to the treatment history and laboratory data, clinical phase of each CHB subject was grouped as previously described into immune active (IA, HBeAg^+^, high viral load and high ALT level), immune tolerant (IT, HBeAg^+^, high viral load but limited liver inflammation), and immune inactive carrier (IC, HBeAg^−^, low or undetectable viral load and normal level of ALT) phases. In addition, 13 acute resolved patients (R, low or undetectable serum viral load, HBsAg-, and HBcAb^+^), 52 patients with liver cirrhosis (LC) and 26 patients with HCC were defined by liver histology or clinical, laboratory and imaging evidences. The exclusion criteria were as follows: patients with hepatitis C virus, hepatitis A virus or human immunodeficiency virus, and malignant tumor. The baseline features (Table 1) and treatment regimens (Table 2) of these patients are presented. Fresh peripheral blood (5 mL) with heparin was collected from each patient during treatment with an interval of 3–5 months. All participants provided written informed consent and Ethics committee approval conforming to the Declaration of Helsinki was obtained from Clinical Ethics Committee of Nanjing Second Hospital (ref: 2021-LS-ky013, 2021-LS-ky014).

**Table 1:** Baseline features of 294 HBV-infected patients enrolled in this study and stratification analyses [Median (Min–Max)]

	Disease stage	n	Age (years)	Gender (M/F)	HBV-specific T cells (SFUs)	ALT (IU/L)	HBV DNA (lg IU/mL)	HBsAg (IU/mL)	HBsAb (mIU/mL)	HBeAg (COI)	HBeAb (COI)	HBcAb (COI)
1	R	13	34 (24–57)	10/3	83 (25–281)	23.9 (11.5–143.8)	< 2.69 (< 2.69–7.41)	< 0.05 (< 0.05)	110.4 (2–704.5)	0.104 (0.081–1.71)	0.021 (0.002–3.47)	0.007 (0.006–0.009)
2	CHB	203	37 (16–71)	147/56	46 (0–622)	29.15 (8.2–391.2)	< 2.69 (< 2.69–8.69)	1374.5 (0.05–52000)	2 (0.52–608.3)	2.51 (0.072–1726)	0.621 (0.002–30.56)	0.007 (0.006–8.44)
3	LC	52	49 (29–72)	41/11	49 (0–285)	30.2 (5.6–295.7)	< 2.69 (< 2.69–3.41)	763.4 (0.2–6487)	2 (2–4.5)	1.16 (0.058–725.6)	0.267 (0.003–3.02)	0.007 (0.006–0.008)
4	HCC	26	54 (25–68)	20/6	93 (0–406)	33.1 (9–214.5)	2.69 (< 2.69–4.415)	212.665 (5.82–2621)	2 (2–35)	0.1125 (0.057–5.43)	0.883 (0.002–1.27)	0.007 (0.006–0.008)
P^*a*^					*p* = 0.0099			*p* < 0.0001	*p* < 0.0001	*p* = 0.0037	*p* = 0.0006	
P^*b*^	R versus CHB				*p* = 0.012			*p* < 0.0001	*p* < 0.0001	*p* = 0.028	*p* = 0.0021	
	R versus LC				*p* = 0.014			*p* < 0.0001	*p* = 0.00042		*p* = 0.0024	
	CHB versus HCC				*p* = 0.026			*p* = 0.0053		*p* = 0.0022	*p* = 0.0022	
	R versus HCC							*p* < 0.0001				
	LC versus HCC				*p* = 0.032							
P^*c*^	*p* = 0.04					*p* = 0.019		*p* = 0.003				

R, acute resolved patients; CHB, chronic hepatitis B; LC, HBV-related liver cirrhosis; HCC, HBV-related hepatocellular carcinoma

ALT (reference values: < 40 IU/L), HBV-DNA (reference values: < 2.69 lg IU/mL), HBsAg (reference values: 0–0.05 IU/mL), HBsAb (reference values: 0–10 IU/mL), HBeAg (reference values: 0–1 COI), HBeAb (reference values: 1–99 COI), HBcAb (reference values: 1–99 COI). COI, cut off index, COI = sample value/cut off value

P^a^, Kruskal–Wallis test (K–W); P^b^, Mann–Whitney test (M–W); P^c^, Multivariate linear regression analysis

**Table 2:** Treatment regimens of 271 treated patients with chronic HBV infection

	Treatment duration before the test time (months)	TMF monotherapy 25 mg QD (patients)	TDF monotherapy 300 mg QD (patients)	TAF monotherapy 25 mg QD (patients)	ETV monotherapy 25 mg QD (patients)	AFD monotherapy 10 mg QD (patients)	TMF/ETV combination 25 mg QD/25 mg QD (patients)
R (NUC)	< 6	9					
CHB (NUCs)	< 3	20	12	11	4		1
	3–12	9	7	7	3	1	2
	12–24	17	10	5	6		
	24–48	9	3	4	6	1	
	> 48	10	3	10	6		
CHB (NUCs/pegIFN)	NUCs: 3–12 pegIFN: < 6 (180 μg QW)	5	6	9	1		
LC (NUCs)	< 3	1		9			
	3–12	2		1			
	12–24	1	2	5	2		2
	24–48	5	2	1	8	1	
	> 48			4	4		
HCC (NUCs)	< 3	1	2				
	3–12			3	3		
	12–24			2	4		
	24–48				1		
	> 48	1		4	2		1

TMF: Tenofovir Amibufenamide; TDF: Tenofovir Disoproxil Fumarate; TAF: Tenofovir alafenamide Fumarate: ETV: Entecavir; AFD: Adefovir Dipivoxil; pegIFN: PEG-interferon-α

### Broad-spectrum HBV CD8^+^ T-cell epitope peptide library

The CD8^+^ T-cell epitopes validated in house were incorporated with the CD8^+^ T-cell epitopes functionally defined by other researchers to establish a HBV-specific peptide library consisting of 105 epitopes [21]. As confirmed by several methods, these epitope peptides were cross-presented by 13 predominant HLA-A allotypes (A1101, A2402, A0201, A0207, A3303, A0206, A3001, A0203, A3101, A1102, A0101, A2601, A0301) which gather a total gene frequency of around 95% in Chinese and Northeast Asian populations [21]. In this study, 103 of the 105 validated epitope peptides (9-mer or 10-mer peptides) were grouped into eight peptide pools according to their derived proteins (HBsAg, HBpol, HBx and HBeAg) and their acidic and alkaline features (Additional file 1: Table S1). Two epitope peptides (P76: FLLAQFTSA; P98: LLAQFTSAI) were excluded because they are the common sequences to the hypothetical protein of Klebsiella Pneumoniae enterobacterial. Then the IFN-γ ELISpot assay was established with the peptide pool array to enumerate the reactive HBV-specific T cells in peripheral blood and followed by the characterization of accuracy and stability in methodology. The HLA-A restrictions of 103 HBV T-cell epitopes used here were exhibited in Additional file 1: Table S2. Their numbers and sequences have already been presented in our recent publication [21]. Meanwhile, we cannot exclude the possibility that these antigenic peptides can also be presented by other HLA-A allotypes beyond the 13 prevalent ones.

### ELISpot assay and detection of HBV-specific T cells

Fresh peripheral blood (5 mL) with heparin was collected from each patient. Peripheral blood mononuclear cells (PBMCs) were isolated by density-gradient centrifugation using Human Lymphocyte Separation Medium (DAKEWE Biotech, Beijing) and freshly used in ELISpot assay.

PBMCs from each patient were seeded into 10 wells (2 × 10^5^ cells/well) in the 96-well ELISpot plates which were pre-coated with human IFN-γ ELISpot capture Antibody (BD Biosciences, 1:200 dilution), and cocultured with eight peptide pools for 20 h in 5% CO_2_ incubator at 37 °C (one well per peptide pool, 2 μg/peptide/well, totally eight experimental wells). In parallel, negative control well (PBMCs alone) and positive control well (PBMCs with phytohemagglutinin, PHA, 2.5 μg/well) were also performed. Notably, in each negative control well, DMSO was supplemented to make its concentration equal to the peptide pool/PBMCs co-culture well. Then, spots were developed with human IFN-γ detection antibody (BD Pharmingen, 1:250 dilution), streptavidin-HRP (BD Pharmingen, 1:100 dilution) and AEC substrate set (BD Pharmingen, 1:50 dilution), according to manufacturer’s protocols. The spot forming units (SFUs) were imaged and enumerated. SFUs in each experimental well = actual spot count in each experimental well—actual spot count in negative control well. When the actual spot count in the indicated experimental well is less than that in the negative control well, SFUs in the indicated experimental well is taken as 0 SFUs. Finally, the total SFUs/2 × 10^5^ PBMCs for eight peptide pools is the sum of SFUs in eight experimental wells. Meanwhile, the SFUs/2 × 10^5^ PBMCs for each HBV protein were also calculated according to the peptide pools derived from each protein. The clinical baseline features and the real-time data from clinical laboratory were also collected at each test time point for each patient.

### Statistical analysis

Statistical analysis was performed using GraphPad Prism 9 (GraphPad, La Jolla, CA, USA). Data were presented as median (interquartile range). A Mann–Whitney (non-parametric) test was used for the analyses of HBV DNA and ALT, SFUs, HBsAg and HBeAg medians between two groups. Kruskal–Wallis test (non-parametric) was performed when analyzing more than two groups. Spearman correlation test was performed to analyze correlation between continuous data. For the analyses between stratified patient groups, multivariate linear regression analysis was performed. The paired student’s *t*-test was performed to compare the dynamic data from the same subjects. Logistic regression analysis was performed, and the diagnostic performances of HBV-specific T cells and sero-virological parameters for predicting liver function progression in CHB patients were assessed by receiver operating characteristic (ROC) and area under the ROC curve (AUC). The DeLong test was performed for the comparison of different ROC curves. *p* < 0.05 was considered statistically significant.

## Results

### HBV-specific T cell reactivity in HBV-infected patients with different clinical profiles

**Fig. 1:**
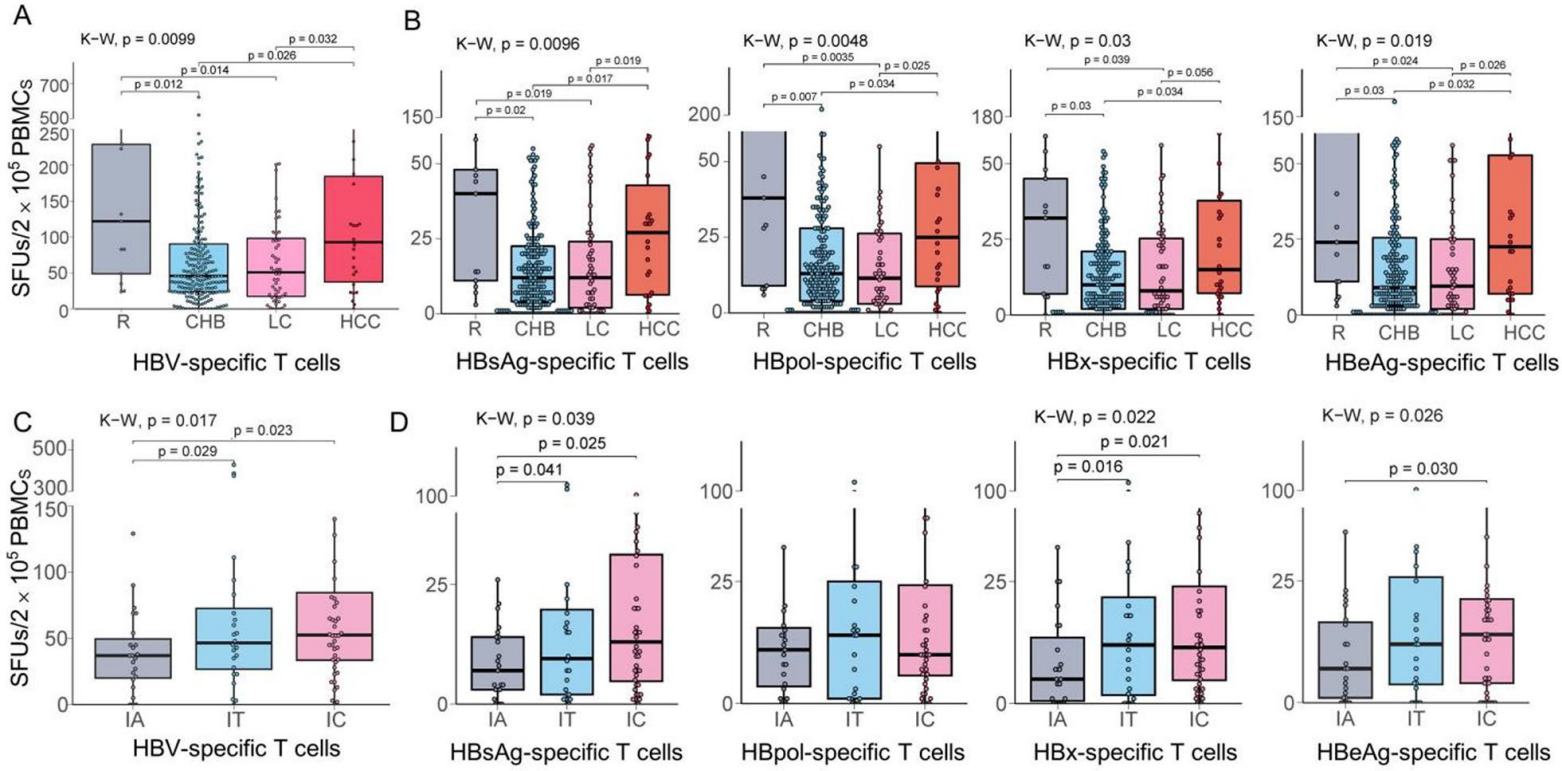
HBV-specific T cell reactivity in 294 HBV-infected patients at different disease stages. Reactive HBV-specific T cells in PBMCs were detected using ex vivo IFN-γ ELISpot assay and 103 validated T-cell epitope peptides. **A** Total HBV-specific T cells (SFUs) in HBV-infected patients at different disease stages (R, n = 13; CHB, n = 203; LC, n = 52; HCC, n = 26). **B** Deconvolution of HBV-specific T cells from total antigens into the indicated HBV protein (HBsAg, HBpol, HBx, HBeAg) in HBV-infected patients.** C** Total HBV-specific T cells (SFUs) in CHB patients at different clinical phases (IA, n = 23; IT, n = 24; IC, n = 44). **D** Deconvolution of HBV-specific T cells from total antigens into the indicated HBV protein (HBsAg, HBpol, HBx, HBeAg) in CHB patients. Medians (interquartile range) were presented and statistical analyses were performed using Kruskal–Wallis test (K–W) across multiple groups and Mann–Whitney test (M–W) between two groups

A total of 294 patients with HBV infection and distinct clinical profiles were detected for the numbers of reactive HBV-specific T cells (SFUs) in PBMCs using the in-house ELISpot assay. As shown in Fig. 1a, acute resolved patients (median 83 SFUs) displayed the highest numbers of reactive HBV-specific T cells while CHB patients (median 46 SFUs) showed the lowest ones among the R, CHB, LC and HCC groups, and an increasing trend of SFU numbers was found from CHB to LC and HCC groups. Meanwhile, the trends of HBsAg-, HBpol-, HBx- or HBeAg-specific T cells across the four disease stages were similar to that of total HBV-specific T cells (Fig. 1b). Moreover, multivariate linear regression analysis was performed for the 294 HBV-infected patients. The SFUs (assigned: continuous variable) were used as the dependent variable, while age, disease stage and sero-virological parameters (all as classification variables) were used as independent variables. The regression equation was tested as F = 4.973, *p* = 0.0009, indicating that the regression model was qualified. The collinearity was evaluated by variance inflation factor (VIF) (ALT: 1.10; DNA: 1.51; HBsAg: 1.42; stages: 1.03). Finally, the number of reactive HBV-specific T cells in PBMCs was significantly correlated with ALT level, HBsAg level, and disease stage (R, CHB, LC and HCC) (Table 1).

Furthermore, the numbers of reactive HBV-specific T cells in PBMCs were compared across the clinical phases of CHB patients. In total, 114 of 203 CHB patients can be grouped into IA, IT or IC phases. A significantly increasing trend of SFU numbers was observed from IA group (median 37 SFUs), IT group (median 53 SFUs) to IC group (median 56 SFUs) with a *p*-value of 0.022 (IA vs. IT) and 0.0093 (IA vs. IC) (Fig. 1c). The trends of HBsAg-, HBx- or HBeAg-specific T cells across the three CHB subgroups were similar to that of total HBV-specific T cells, but HBpol-specific T cells displayed no significant differences across the CHB subgroups (Fig. 1d). In each CHB phase, the numbers of reactive HBV-specific T cells induced by different HBV proteins showed no significant difference (Additional file 1: Fig. S1). The spot plots reactive to each peptide pool in the in-house ELISpot assay were presented for five representative CHB subjects (Additional file 1: Fig. S2).

### HBV-specific T cell reactivity in CHB patients with different sero-virological profiles

**Fig. 2:**
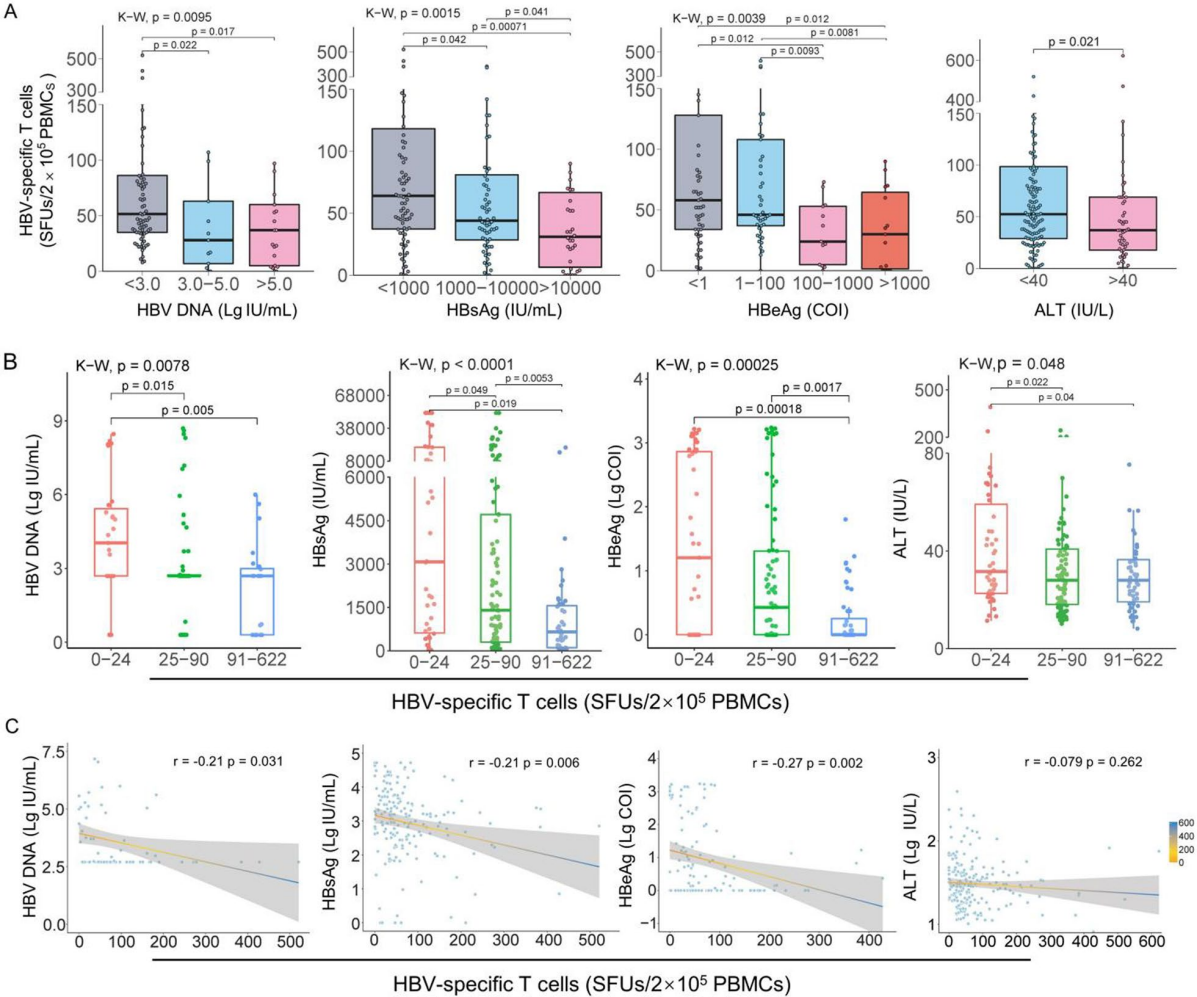
Association of HBV-specific T cell reactivity with sero-virological parameters in CHB patients. **A** Stratified analyses of HBV-specific T cells (SFUs) in CHB patients grouped by HBV DNA load (< 3.0, n = 70; 3.0–5.0, n = 27; > 5.0, n = 23), HBsAg level (< 1000, n = 82; 1000–10000, n = 64; > 10,000, n = 34), HBeAg level (< 1, n = 53; 1–100, n = 45; 100–1000, n = 13; > 1000, n = 15) and ALT level (< 40, n = 146; > 40, n = 56). **B** Stratified analyses of sero-virological parameters in CHB patients grouped by HBV-specific T cell reactivity (0–24 SFUs for 25% of the cohort; 25–90 SFUs for 50% of the cohort; 91–622 SFUs for 25% of the cohort). For HBV DNA load, HBsAg level, HBeAg level, and ALT level analyses, 0–24 group, n = 42, 27, 41, 50, respectively; 25–90 group, n = 93, 59, 77,101, respectively; 91–622 group, n = 45, 24, 52, 51, respectively. **C** Spearman correlation tests between HBV-specific T cells (SFUs) and HBV DNA, HBsAg, HBeAg or ALT levels

Stratified analyses manifested that the numbers of reactive HBV-specific T cells in PBMCs of CHB patients presented a significantly declined trend when the serum HBV DNA load, HBsAg, HBeAg or ALT level gradually increased (Fig. 2a). Considering different treatments may bring impact on the HBV-specific T cell reactivity, the stratified analyses were repeated in a cohort of CHB patients undergoing NUCs monotherapy (n = 167). The results (Additional file 1: Fig. S3) were in accordance with those from 203 CHB patients who were undergoing different therapies (NUCs alone or combined with IFN-α) (Fig. 2a). Furthermore, the numbers of HBsAg-, HBpol-, HBx-, or HBeAg-specific T cells also showed a decreasing trend similar to that of total HBV-specific T cells when HBV DNA, HBsAg, HBeAg and ALT levels gradually increased (Additional file 1: Fig. S4).

To further assess the association of HBV-specific T cell reactivity with sero-virological parameters, CHB patients (n = 203) were divided into three subgroups according to SFU levels by using interquartile method: 25% of the cohort who presented low SFU level (0–24 SFUs/2 × 10^5^ PBMCs), 50% of the cohort who presented intermediate SFU level (25–90 SFUs/2 × 10^5^ PBMCs), and 25% of the cohort who presented high SFU level (91–622 SFUs/2 × 10^5^ PBMCs). As shown in Table 3 and Fig. 2b, serum HBV-DNA, HBsAg, HBeAg and ALT levels were the highest in the subgroups with low SFU level, and the lowest in the subgroup with high SFU level.

**Table 3:** Stratification analyses of HBV-specific T cell reactivity for 203 CHB patients

	ELISpot (SFUs)	HBV DNA median (min–max)	n	HBsAg (IU/ml) median (min–max)	n	HBeAg (COI) median (min–max)	n	ALT (IU/L) median (min–max)	n
low	0–24	4.04 (0.3–7.04)	27	3075 (0.05–8895)	42	47.19 (0.079–158.5)	30	31.6 (11.4–391.2)	50
inter	25–90	2.69 (0.3–8.69)	58	1474.5 (0.05–52000)	91	3.7 (0.073–1726)	64	28.7 (10.2–243.4)	99
high	91–622	2.69 (0.3–5.99)	24	652.8 (0.05–20428)	45	0.336 (0.072–63.96)	30	28 (8.2–105.6)	51
*Statistics*									
P^*a*^		*p* = 0.0078		*p* < 0.0001		*p* = 0.00025		*p* = 0.048	
P^*b*^	Low versus inter	*p* = 0.015		*p* = 0.049				*p* = 0.022	
	Low versus high	*p* = 0.005		*p* = 0.0053		*p* = 0.00018		*p* = 0.04	
	Inter versus high			*p* = 0.0053		*p* = 0.00017			
P^*c*^		*p* = 0.34638		*p* = 0.00723				*p* = 0.16839	

HBV-DNA (reference values: < 2.69 lg IU/mL), HBsAg (reference values: 0–0.05 IU/mL), HBeAg (reference values: 0–1 COI; COI, cut off index, COI = sample value/cut off value), ALT (reference values: < 40 IU/L)

P^a^, Kruskal–Wallis test (K–W); P^b^, Mann–Whitney test (M–W); P^c^, Multivariate linear regression analysis

### Correlation between HBV-specific T cell reactivity and sero-virological parameters in CHB patients

Multivariate linear regression analysis was performed for the 203 CHB patients (F = 3.384, *p* = 0.021). VIF was applied for multicollinearity (DNA load: 1.74; HBsAg: 1.43; ALT: 1.28) and showed a weaker degree of multicollinearity in the model. The *p* values of DNA, ALT, and HBsAg items were 0.168, 0.346 and 0.007, respectively (Table 3). That means the numbers of reactive HBV-specific T cells in PBMCs of CHB patients only significantly correlated with HBsAg level. Moreover, age, HBeAg, different phases of CHB (IT, IA and IC) were also used as independent variables in different regression models, but no correlation with SFU levels was found.

Furthermore, Spearman correlation tests between the numbers of reactive HBV-specific T cells in PBMCs and each sero-virological parameter were conducted for the 203 CHB patients. The SFU numbers were weakly and negatively correlated with serum HBV DNA (r = − 0.21), HBsAg (r = − 0.21) and HBeAg (r = − 0.27) levels, but not ALT level (r = − 0.079) (Fig. 2c). Meanwhile, HBsAg-, HBpol-, HBx-, or HBeAg-specific T cells also negatively correlated with serum HBV DNA, HBsAg and HBeAg levels with a very low coefficient (r = − 0.12 to − 0.26), and not correlated with ALT levels (r = − 0.027 to − 0.12) (Fig. S5). In addition, the correlations tests were repeated for the IA-phase, IT-phase and IC-phase patients, respectively, and no significant correlations between the SFU numbers and each sero-virological parameter was found in each subgroup (Additional file 1: Fig. S6).

### Association of HBV-specific T cell reactivity with anti-virus therapy

**Fig. 3:**
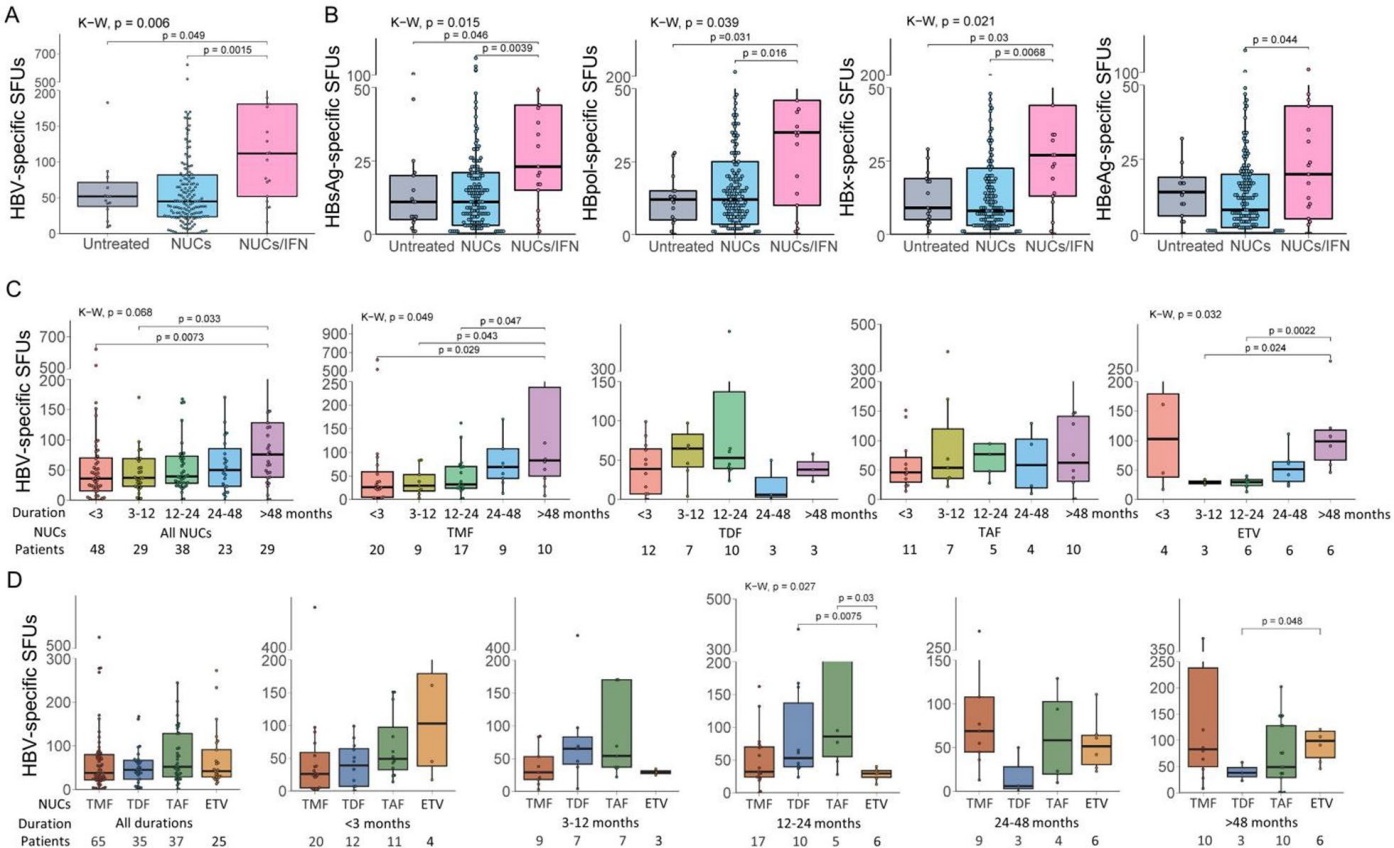
Association of HBV-specific T cell reactivity with anti-virus therapy in CHB patients. **A** HBV-specific T cells (SFUs) in CHB patients with different treatments. **B** Specific T cells (SFUs) reactive to each HBV protein (HBsAg, HBpol, HBx, HBeAg) in different treatment groups. Untreated group, n = 15; NUCs monotherapy, n = 167; NUCs/IFN combination therapy, n = 21. **C** HBV-specific T cells (SFUs) after NUCs treatment in different durations of treatment. **D** HBV-specific T cells (SFUs) after different NUCs treatment in the same treatment duration. Medians (interquartile range) were presented and statistical analyses were performed using Kruskal–Wallis test (K–W) across multiple groups and Mann–Whitney test (M–W) between two groups

HBV-specific T cells were further investigated and compared between the NUCs treatment group and NUCs/IFN-α combination group for CHB patients. The numbers of reactive HBV-specific T cells in NUCs/IFN-α group (median 103 SFUs) were obviously higher than those in the untreated (median 53 SFUs) or NUCs monotherapy (median 45 SFUs) groups (Fig. 3a). HBsAg-, HBpol-, HBx- or HBeAg-specific T cells also displayed the trends similar to total HBV-specific T cells across groups (Fig. 3b). Obviously, NUCs/IFN-α combination led to much more reactive HBV-specific T cells than NUCs monotherapy in CHB patients. In different treatment duration subgroups, the NUCs treatment more than 4 years presented more reactive HBV-specific T cells than the NUCs treatment less than 1 year, especially TMF treatment (Fig. 3c), but different NUCs in the same duration of treatment did not bring different reactivity of HBV-specific T cells (Fig. 3d).

### Dynamic reactivity of HBV-specific T cells in CHB patients with different sero-virological courses

**Fig. 4:**
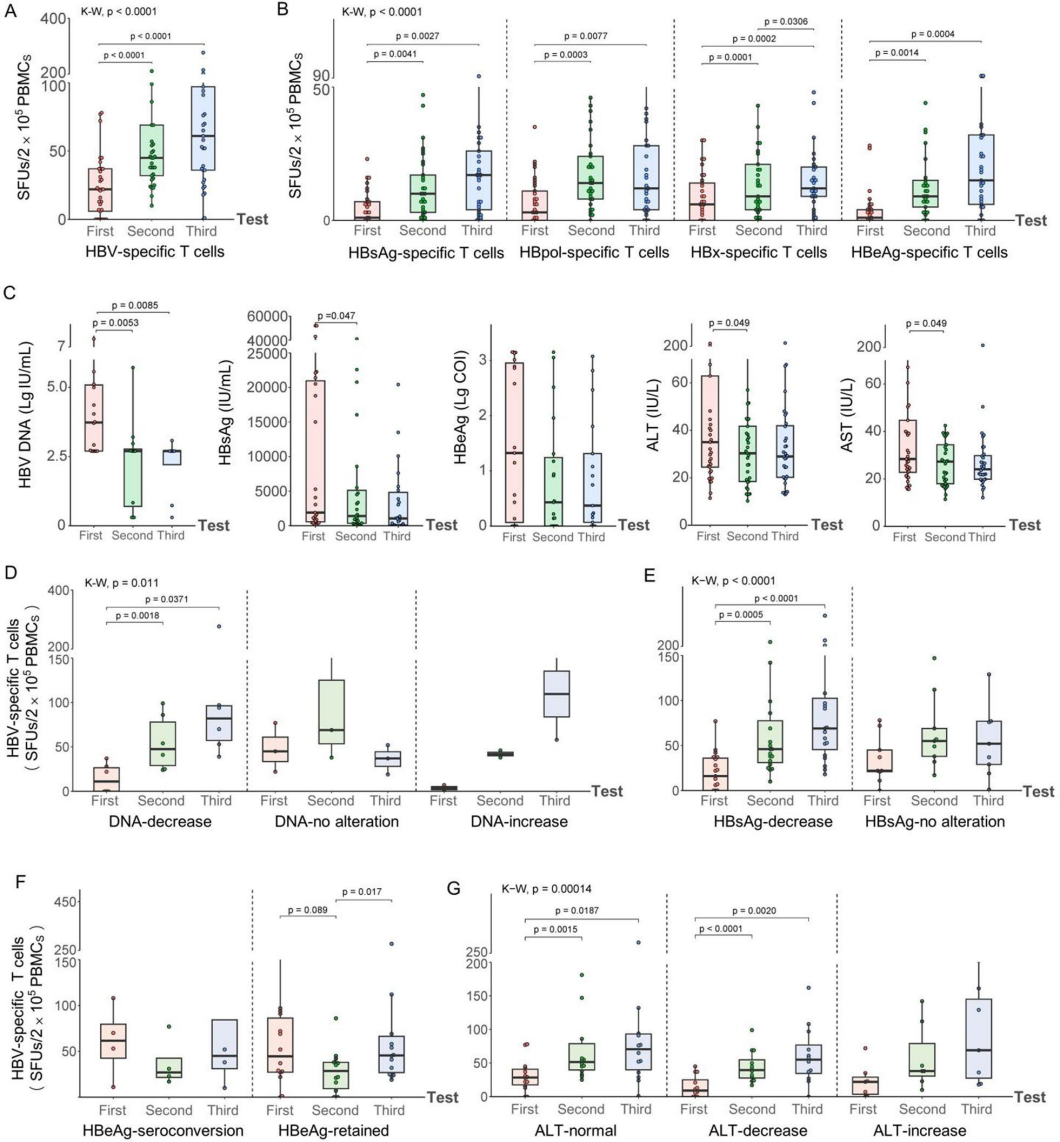
Dynamic changes of HBV-specific T cells and sero-virological parameters in CHB patients. 33 CHB patients undergoing routine treatment were followed by HBV-specific T cell detection and sero-virological parameters collections for three times at an interval of 3–5 months. **A**, **B** Dynamic changes of total HBV-specific T cells and the specific T cells reactive to each HBV protein in 33 CHB patients. **C** Dynamic changes of HBV DNA (n = 18), HBsAg (n = 27), HBeAg (n = 19), ALT (n = 32), and AST (n = 32) levels. Then, the dynamic changes of HBV-specific T cells in CHB patients with different fluctuation courses of** D** HBV DNA load (decrease, n = 7; no alternation, n = 3; increase, n = 2),** E** HBsAg level (decrease, n = 18; no alternation, n = 10), **F** HBeAg level (seroconversion, n = 4; retained, n = 13), and **G** ALT level (normal, n = 14; decrease, n = 12; increase, n = 7) were presented. The patients who achieved DNA fluctuations (increase or decrease) > 30% were defined as the DNA-increase or DNA-decrease group, and the other patients were defined as DNA-no alternations group. HBsAg-decrease was defined as an amplitude decrement of more than 30%. CHB patients who experienced a positive HBeAg serology (HBeAg COI > 1) at first and seroconverted (HBeAg COI < 1) later were defined as the HBeAg-seroconversion group. ALT-decrease was defined as a decline to the normal range (< 40 IU/L) or decreased more than 30%, while ALT that rose more than 30% or beyond 40 IU/L was defined as ALT-increase. The paired, two-tailed Student’s t tests between two groups and Kruskal–Wallis test (K–W) across more than two groups were performed

HBV-specific T cells were detected three times at an interval of 3–5 months for 33 CHB patients who undergoing NUCs monotherapy or NUCs/IFN-α combination therapy, and sero-virological data were synchronously collected at the same time point. The treatment regimens and duration before the first test were presented in Additional file 1: Table S3. The numbers of reactive HBV-specific T cells were obviously and gradually increasing during the three tests (Fig. 4a). HBsAg-, HBpol-, HBx-, and HBeAg-specific T cell responses were consistent with the total HBV-specific T cell responses (Fig. 4b). Meanwhile, serum HBV DNA loads, HBsAg, HBeAg and ALT/AST levels displayed a continuously declined tendency during the observation period (Fig. 4c).

Subsequently, the 33 CHB patients were divided into the different subgroups with an increasing, no-alteration or declining sero-virological course, and stratified analysis were further performed. As displayed, the numbers of reactive HBV-specific T cells continuously increased from baseline (first test) to third test in the DNA load decreasing subgroup, but no significant increase in the no-alteration subgroup or increasing subgroup (Fig. 4d). Similarly, the SFU numbers also continuously increased in the HBsAg level decreasing subgroup, while no significant increase in the no-alteration subgroup (Fig. 4e). Notably, in the HBeAg-retained subgroup, SFU numbers exhibited a trend of initially decreasing and subsequently increasing, while no meaningful pattern was discernible in the HBeAg seroconversion group (Fig. 4f). Meanwhile, the SFU numbers exhibited a significant increase in ALT normal or decreased subgroups (Fig. 4g).

To confirm the dynamic trends in the absence of IFN-α treatment, the longitudinal data were collected from 28 CHB patients who only undergoing NUCs monotherapy, then the dynamic tendency and conclusion were obtained (Additional file 1: Fig. S7) and as same as that in Fig. 4.

### Dynamic tendencies of sero-virological parameters in CHB patients with different HBV-specific T cell courses

**Fig. 5:**
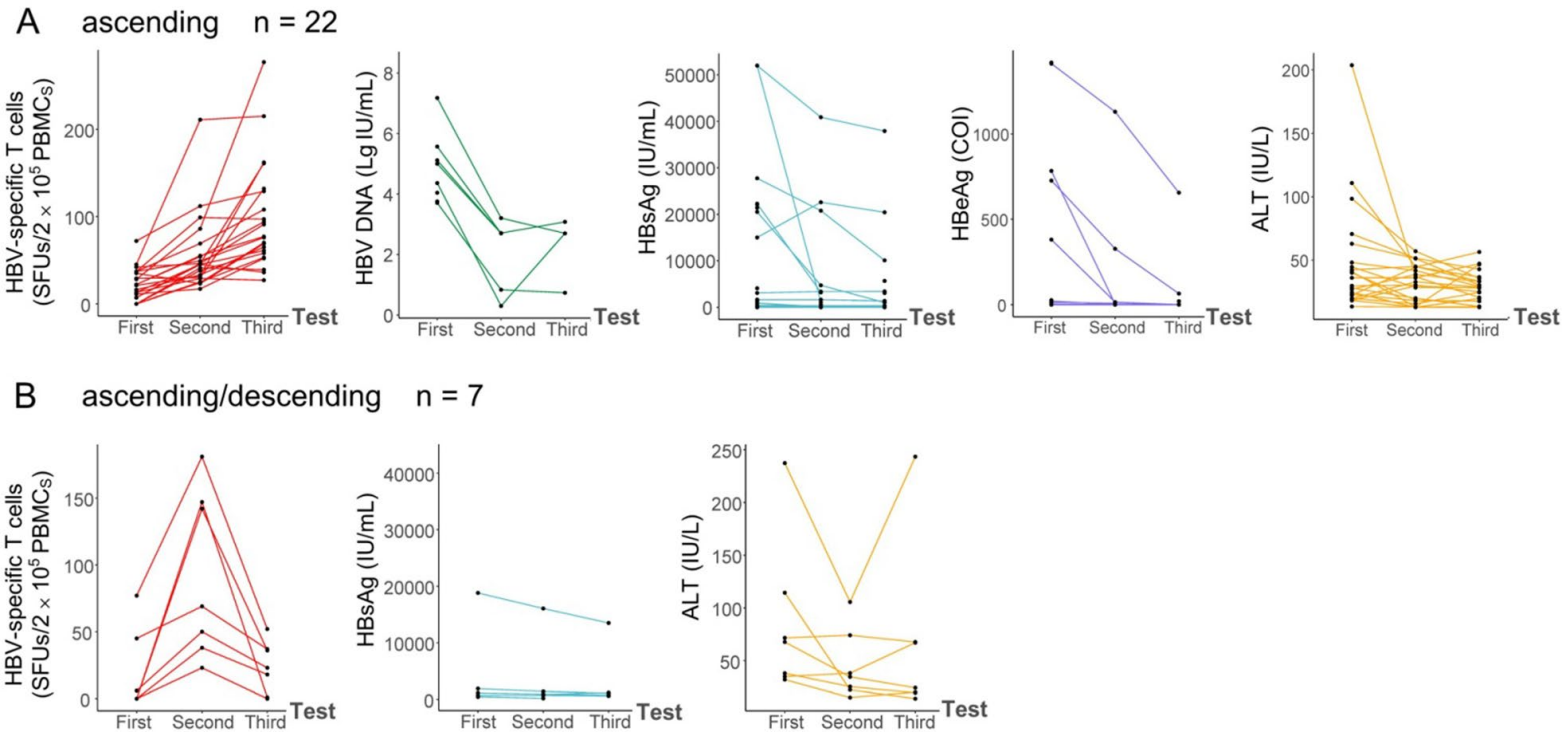
Dynamic changes of sero-virological parameters during different fluctuation courses of HBV-specific T cells. 33 CHB patients were followed by HBV-specific T cell detection and sero-virological parameters collections for three times at an interval of 3–5 months. According to the dynamic courses of HBV-specific T cells (SFUs), patients were categorized as ascending (**A**), ascending/descending (**B**), stationary, descending, or descending/ascending groups, then HBV DNA load, HBsAg, HBeAg, HBx, HBpol and ALT levels were longitudinally analyzed. The SFU numbers of reactive HBV-specific T cells which increased or decreased more than 50% than last test were defined as ascending or descending during the follow-up period

Here, the 33 CHB patients was categorized into several subgroups according to the different courses of HBV-specific T cell reactivity during the three tests. The fluctuation (increase or decrease) of about 50% of SFU numbers was defined as an obvious change between two tests, thus 5 subgroups were obtained: ascending, ascending/descending, stationary, descending, and descending/ascending. The dynamic spot plots of ELISpot assay for representative ascending patients and ascending/descending patient were presented in Additional file 1: Fig. S8. As expected, in the SFU numbers ascending subgroup (n = 22), a continuous decrease of HBV-DNA loads, HBsAg levels, HBeAg levels, or ALT levels was found in most cases (Fig. 5a). In the SFU numbers ascending/descending subgroup (n = 7), HBsAg levels and ALT levels retained stable or slight decline in most cases (Fig. 5b). The dynamic courses in other subgroups cannot be concluded due to small cohorts.

### Predictive power of cross-sectional and longitudinal numbers of reactive HBV-specific T cells for liver function progression in CHB patients

**Fig. 6:**
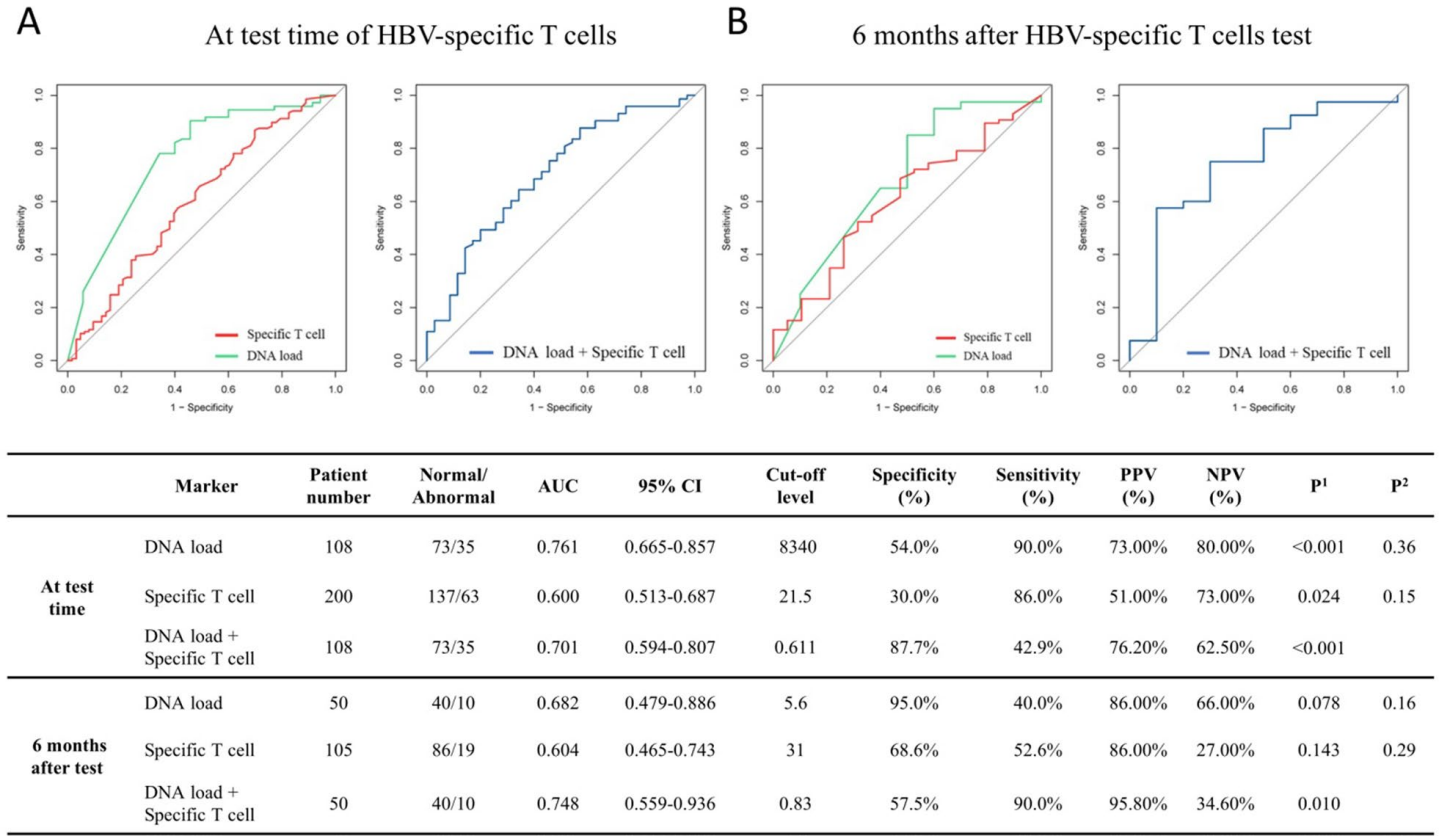
Predictive power of cross-sectional reactivity of HBV-specific T cells for hepatitis progression in CHB patients. CHB patients were divided into normal (ALT < 40 IU/L) group and abnormal (ALT > 40 IU/L) group of liver function at the test time of HBV-specific T cells or 6 months later after the test. ROC curve analyses of DNA load (IU/mL), HBV-specific T cells (SFUs/2 × 10^5^ PBMCs), and a combination were performed to predict hepatitis progression at the test time of HBV-specific T cells (**A**) and 6 months later after the test (**B**), using R package pROC, and summarized in table. ROC, receiver operating characteristic; AUC, area under the curve; PPV, positive predictive value; NPV, negative predictive value. The *p*^1^ values represent the significance of model. The *p*^2^ values represent the significance of difference between the AUC of combined markers (DNA load + Specific T cell) and single predictor

Firstly, the predictive power of cross-sectional numbers of reactive HBV-specific T cells from CHB patients were evaluated for the hepatitis progression at test time and 6 months later. As shown in Fig. 6, HBV-specific T cells presented 0.600 AUC, 21.5 (SFUs) cut-off value, 30.0% specificity and 86.0% sensitivity for the liver function at test time, and 0.604 AUC, 31 (SFUs) cut-off value, 68.6% specificity, and 52.6% sensitivity for the liver function 6 months later. Comparably, HBV DNA load presented a higher predictive value. Further, ROC curves for the combined two factors conferred a further increase in predictive accuracy (0.748 AUC, 57.5% specificity, 90.0% sensitivity) at 6 months after T-cell test. These data suggest that the cross-sectional number of reactive HBV-specific T cells, as a parameter of host adaptive immunity, is also a valued predictor for liver function progression in CHB patients undergoing routine treatment, especially when combined with the viral DNA load. Then, the data of HBV-specific T cells (SFUs/4 × 10^5^ PBMCs) and sero-virological parameters were collected from another 176 CHB patients (beyond the CHB patients in Fig. 6), and were used to confirm the predictive power by ROC analysis. As shown in Additional file 1: Fig. S9, the AUC values of SFU numbers were much higher than that in Fig. 6 (0.750 vs. 0.600 at test time; 0.662 vs. 0.604 at 6 months later). Meanwhile, viral DNA load did not present a higher predictive value than HBV-specific T cells and the combined two factors only presented a slightly increased predictive accuracy (0.722 vs. 0.662 AUC at 6 months after T cell test).

**Fig. 7:**
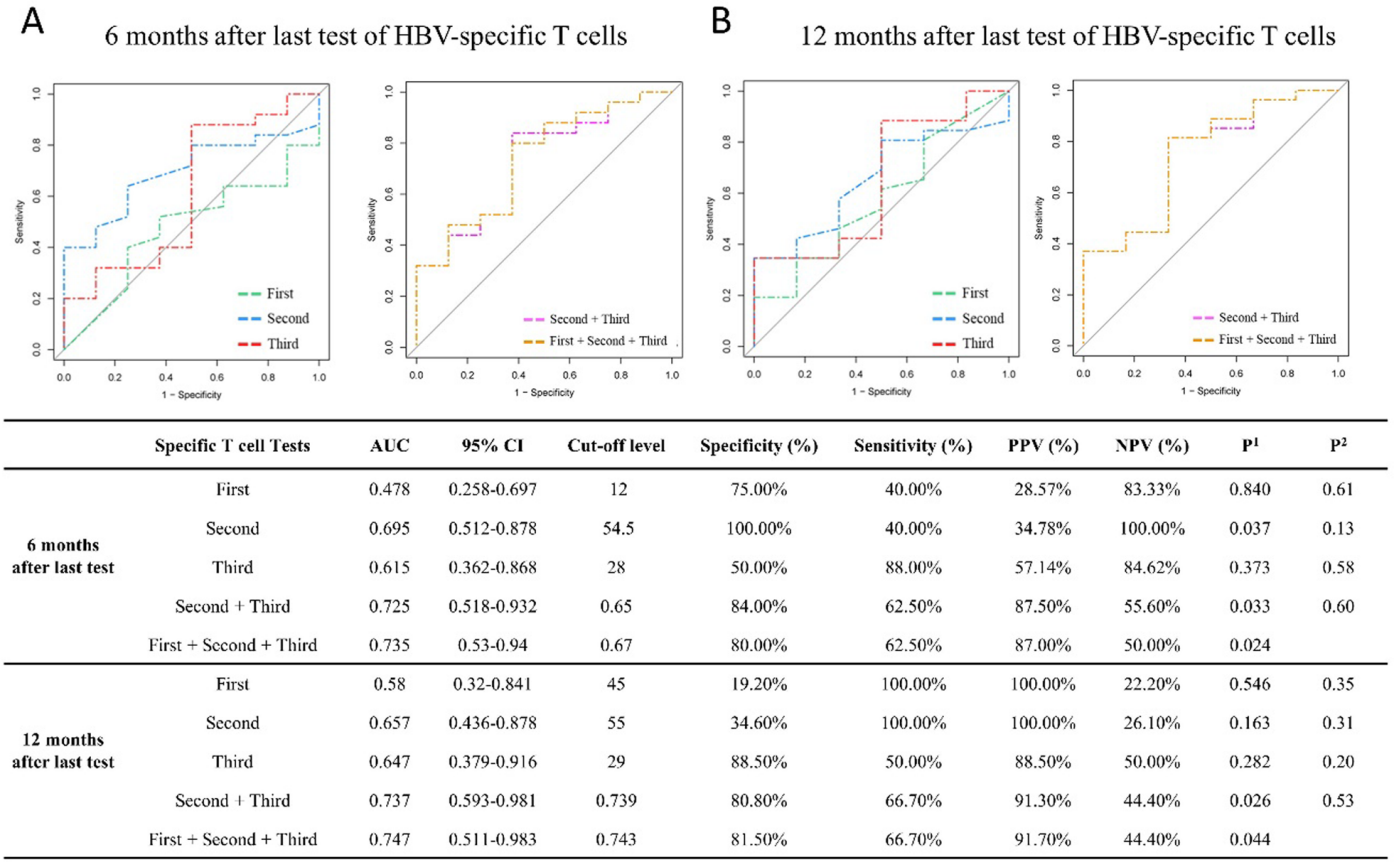
Predictive power of longitudinal reactivity of HBV-specific T cells for liver hepatitis progression in CHB patients. 33 CHB patients undergoing NUCs or NUCs/INF-α treatment were divided into normal (ALT < 40 IU/L) group and abnormal (ALT > 40 IU/L) group of liver function at 6 months (normal/abnormal: 8/25) or 12 months (normal/abnormal: 6/27) after the last test of HBV-specific T cells. ROC curve analyses of single test and combined tests of HBV-specific T cells (SFUs/2 × 10^5^ PBMCs) were performed to predict hepatitis progression at 6 months (**A**) and 12 months after the last test (**B**) of HBV-specific T cells, using R package pROC, and summarized in table. The *p*^1^ values represent the significance of model. The *p*^2^ values represent the significance of difference between the AUC of combined markers (First + Second + Third) and other predictors

Additionally, ROC curve analyses were also performed on the longitudinal data of reactive HBV-specific T cells from the 33 CHB patients who were tested three times at an interval of 3–5 months. When compared to when either was analyzed independently, the combination of first, second and third test led to the highest AUC value to predict liver function progression at 6 months or 12 months after the last test. The AUC was 0.735 and 0.747, specificity was 80.0% and 81.5%, and sensitivity was 62.5% and 66.7%, respectively (Fig. 7). These data imply that the longitudinal monitoring of HBV-specific T cell reactivity possesses much higher predictive power for hepatitis progression than the cross-sectional detection.

## Discussions

Unlike the routine tests of antigens and antibodies, the clinical detection of antigen-specific T cells is markedly limited by the high polymorphisms of HLA allotypes in patient cohort. Currently, peptide/HLA (pHLA) tetramer or multimer staining is the most specific technique for antigen-specific T cell detection since it was first reported in 1996, but the preparation of pHLA multimer is time-consuming and expensive. Therefore, it is unfeasible to prepare a library containing hundreds of pMHC multimers using numerous predominant HLA subtypes and numerous T-cell epitope peptides for the routine test of an indicated disease in clinical laboratory. In research laboratory, an alternative and wide-used way is the co-cultures of antigen-derived T-cell epitope peptides with patient’s peripheral lymphocytes followed by an enumeration of cytokine-secreted cells using ELISpot assay, in which one cytokine-secreting cell can be detected among one million cells, a much higher sensitivity than pHLA multimer staining [22]. In order to facilitate the routine test of functional HBV-specific T cells in clinics, this study chose a library of CD8^+^ T-cell epitope peptides (9–10-mer) that not only can target as many as 103 HBV-specific CD8^+^ T cell clones specific for HBsAg, HBeAg, HBx or HBpol proteins, but also fits to the herd HLA genetic characteristics of Chinese and Northeast Asian populations. More importantly, they are functionally validated CD8^+^ T-cell epitopes using blood samples from around 700 CHB patients as described previously [21]. Using the broad-spectrum library of T-cell epitope peptides, an ELISpot assay was established in clinical laboratory. Then the reproducibility or accuracy in methodology was evaluated and a low intra-assay and inter-assay CV (7.26%, 7.95%) was achieved (Additional file 1: Table S4, Additional file 1: Fig. S10). Totally, 294 random HBV-infected patients including 203 CHB patients were investigated for cross-sectional or longitudinal studies in this study, and five conclusions can be drawn that are comparable with the outcomes of previous reports.

First conclusion, functional HBV-specific T cells (mainly CD8^+^ T cells since the use of 9–10-mer peptides) in PBMCs displayed an increased trend from CHB, LC, HCC to R phases (no statistical difference between LC and HCC), and also from IA, IT to IC phases of CHB (no statistical difference between IT and IC). HBsAg-, HBpol-, HBx- or HBeAg-specific T cells also maintained the similar tendency. Similarly, Boni et al. [23] and Hoogeveen et al. [12] also reported higher frequencies of functional HBV-specific T cells in R patients than CHB patients as detected by in vitro ICS or ex vivo ELISpot using 15-mer or 18-mer OLPs covering the four proteins. Controversially, Brinck-Jensen et al. reported the similar SFU numbers of HBsAg-specific or HBcAg-specific T cells between 20 CHB patients and 20 R patients as detected by in vitro ELISpot using 10 functionally validated and 10 in silico predicted CD8^+^ T-cell epitope peptides [14]. Very few studies have further reported the changes of functional HBV-specific CD4^+^ or CD8^+^ T cells from CHB to LC or HCC stages. For IA, IT and IC phases in CHB patients, Chen reported the increasing HBV-specific T cell activity from IA, IT to IC phases in 60 CHB patients by in vitro ELISpot assay using 15-mer OLPs from the four proteins, but no significant difference between IA and IC [11]. Our data showed no difference between IT and IC phases. More importantly, previous studies have documented that HBcAg induced stronger specific T cell response than HBeAg and HBx protein in CHB patients, especially in IC patients, as detected by in vitro ELISpot assay using 15-mer OLPs [11] or 18-mer OLPs [24] covering HBV proteome. However, our data showed no statistical difference in the numbers of specific T cells (mainly CD8^+^ T cells) reactive to different HBV proteins in each disease stage, including R, CHB, LC, HCC, IA, IT, or IC stages.

Second conclusion, although the numbers of functional HBV-specific T cells (mainly CD8^+^ T cells) in PBMCs strongly and negatively associated with sero-virological parameters (viral DNA load, HBsAg, HBeAg, and ALT) in CHB patients as confirmed by stratified analyses, but only a very low correlation coefficient (r = − 0.21, − 0.21, − 0.27, − 0.079, respectively) was defined by Spearman correlation tests. Even more, no correlation was found in IA, IT and IC subgroups. HBsAg-, HBpol-, HBx- or HBeAg-specific T cells also displayed the similar associations. Based on the conflicting results between stratified analyses and Spearman correlation tests, we speculate that HBV-specific T cell reactivity is determined mainly by host immune defense function rather than viral DNA load and antigen level. Previously, numerous contradictory results were also reported. For example, HBcAg-specific T cells negatively correlated with viral load and HBsAg level in CHB patients [11]; HBsAg-specific T cells positively correlated with HBsAg level, but multivariable linear regression models identified the age of patients as the only significant covariate associated with the HBsAg-specific T cells [10]; No correlation was found between HBsAg level and HBsAg-specific CD4^+^ T cells and CD8^+^ T cells in 57 CHB patients [13]. Our data indicated that the functional HBV-specific T cells significantly correlated with HBsAg and ALT levels and disease stage (R, CHB, LC, HCC) in 294 HBV-infected patients, but only correlated with HBsAg level in 203 CHB patients as defined by multivariate linear regression analyses. Presumably, these contradictory results mainly contributed by the diverse OLPs, detection methods, patient cohorts, and different T cell groups (CD4^+^ or CD8^+^ T cells were detected).

Third conclusion, ROC analyses suggest the longitudinal numbers of reactive HBV-specific T cells in PBMCs is a valued predictor for hepatitis progression 6 or 12 months later in the CHB patients undergoing routine anti-viral treatment. T cells may be a double-edged sword for liver function because they can kill virus-infected liver cells and inhibit virus replication, but also cause liver inflammation [25]. The causal relationship between T cell reactivity and hepatitis is still difficult to explain clearly, and our data initially reported the predictive value of HBV-specific T cells for hepatitis in CHB patients.

Fourthly, the current available data on T cell responses related to CHB patients mostly came from cross-sectional studies [1, 10, 16, 23, 26–29], longitudinal analyses for functional HBV-specific T cells, especially during the different course (decreasing, no alteration, or increasing) of DNA loads, HBsAg levels, HBeAg levels, ALT levels and NUCs or pegIFN-a treatment is still lacked. Our data initially concluded that the numbers of reactive HBV-specific T cells in PBMCs were obviously increasing in the CHB patients undergoing anti-viral treatment and came along with the continuous decline of serum viral DNA load, HBsAg and HBeAg level, and along with the gradual decrease of ALT level. These longitudinal results of HBV-specific T cell reactivity in different sero-virological courses and the dynamic data of sero-virological parameters in the continuously ascending course of HBV-specific T cell reactivity may be able to stop the disputes about the correlations of HBV-specific T cells with sero-virological parameters in cross-sectional studies.

Finally, whether NUCs or IFN-α monotherapy can enhance HBV-specific T cell response remains disputed [1, 10, 16, 27–30]. Our data indicated that CHB patients undergoing NUCs/pegIFN-α combination therapy displayed much more reactive HBV-specific T cells than the patients undergoing NUCs monotherapy, and HBsAg-, HBpol-, HBx- or HBeAg-specific T cells also maintained the similar tendency. Meanwhile, our longitudinal analyses confirmed that both NUCs monotherapy and NUCs/pegIFN-α combination therapy obviously increased the functional HBV-specific T cells in PBMCs.

## Conclusions

In contrast to previous studies, the routine tests of functional HBV-specific T cells have been initially established in clinical laboratory using broad-spectrum and validated T-cell epitope peptides rather than OLPs or PEPs. Although the patient cohort and detection technique probably influence the outcomes, our data more authentically recapitulates the cross-sectional pattern and longitudinal characteristics of HBV-specific T cell reactivity and their associations with NUCs/pegIFN-α treatment and sero-virological parameters in Chinese patients. The presented method could be developed into an efficient reference method for the clinical evaluation of HBV-specific cellular immunity. The patients presenting low reactivity of HBV-specific T cells have a worse prognosis for hepatitis progression and should be treated using pegIFN-α to improve host T-cell immunity.

### Supplementary Information

**Additional file 1.** Supplementary materials, tables and figures.

## Data Availability

The datasets used and/or analysed during the current study are available from the corresponding author on reasonable request.

## Abbreviations

HBV: Hepatitis B Virus
LC: Liver cirrhosis
HCC: Hepatocellular carcinoma
CHB: Chronic hepatitis B
HLA: Human leukocyte antigens
ELISpot: Enzyme-linked immunosorbent spot
ICS: Intracellular cytokine staining
PBMCs: Peripheral blood mononuclear cells
ALT: Alanine aminotransferase
AST: Aspartate aminotransferase
PHA: Phytohemagglutinin
SFUs: Spot forming units
IA: Immune active
IT: Immune tolerant
IC: Immune inactive carrier
OLPs: Overlapping peptides
PEPs: In silico predicted T-cell epitope peptides
HBsAg: Hepatitis B surface antigen
HBcAg: Hepatitis B core antigen
HBeAg: Hepatitis B e antigen
HBpol: Hepatitis B polymerase protein
HBx: Hepatitis B X protein
NUCs: Nucleotide Analogs
ROC: Receiver operating characteristic
AUC: Area under the ROC curve
K–W: Kruskal–Wallis test
M–W: Mann–Whitney test
