# Supplementary material for: Routine evaluation of HBV-specific T cell reactivity in chronic hepatitis B using a broad-spectrum T-cell epitope peptide library and ELISpot assay
Source: J Transl Med. 2024 Mar 11;22:266. doi: 10.1186/s12967-024-05062-5 (PMC10929206; doi:10.1186/s12967-024-05062-5)
Supplement: Supplementary file 1 — Additional file 1. Supplementary materials, tables and figures. [file 12967_2024_5062_MOESM1_ESM.pdf]

Supplementary materials

Supplementary Tables

Table S1 Peptide pools of 103 T-cell epitopes of HBV antigens in the in-house ELISpot assay

Peptide pool	Pool 1	Pool 2	Pool 3	Pool 4	Pool 5	Pool 6	Pool 7	Pool 8
**Derived protein**	HBsAg	HBsAg	HBpol	HBpol	HBx	HBx	HBeAg	HBeAg
**Kinds of peptide**	17	15	18	8	13	7	15	10

Table S2 Antigen distribution and HLA-A restrictions of 103 T-cell epitopes of HBV antigens in the clinical detection of HBV-specific T cells

HLA-A allotype	Allele frequency	HBV $CD8^+$ T-cell epitope peptides				Total
		HBsAg	HBeAg (covering HBcAg)	HBpol	HBx	
A1101	22.4%	10	8	9	6	33
A2402	15.3%	9	9	6	4	28
A0201	13.9%	9	11	9	4	33
A0207	9.5%	7	9	3	1	20
A3303	8.1%	6	3	4	2	15
A0206	6.1%	2	4	5	2	13
A3001	5.2%	2	3	3	4	12
A0203	3.9%	3	6	6	2	17
A3101	3.4%	3	2	2	3	10
A1102	2.3%	8	5	3	5	21
A0101	2.2%	4	1	2	1	8
A2601	1.8%	2	2	3	1	8
A0301	1.4%	6	3	2	4	15
Total	95.5%	32	25	26	20	103

Table S3 Treatment regimens of 33 CHB patients for the longitudinal detection of HBV-specific T cells

	Treatment duration before the first test	TMF 25 mg QD (patients)	TDF 300 mg QD (patients)	TAF 25 mg QD (patients)	ETV 25 mg QD (patients)
**CHB (NUCs monotherapy)**	< 3 months	7	6	2	
	3-12 months			1	
	12-24 months	4			1
	24-48 months	1	3	1	
	>48 months	1		2	
**CHB (NUCs/pegIFN)** pegIFN: 180 μg QW	NUCs: < 3 months pegIFN: < 2 weeks	1	1	1	
	NUCs: 24-48 months pegIFN: < 2 weeks			1	

Table S4 Intra-assay and inter-assay CV values of SFUs in the in-house ELISpot assay

	Patient	First test (SFUs)	Second test (SFUs)	Third test (SFUs)	Mean (SFUs)	SD	CV (%)	Mean CV (%)
Intra-assay	Patient 1	43	46	44	44	1.52	3.44	7.26
	Patient 2	160	134	142	145	13.3	9.16	
	Patient 3	258	303	284	281	22.5	8.02	
	Patient 4	9	10	9	9	0.57	6.18	
	Patient 5	53	44	47	48	4.58	9.50	
Inter-assay	Patient 6	10	12	9	10	1.52	4.78	7.96
	Patient 7	214	239	239	230	14.4	6.25	
	Patient 8	316	283	365	321	41.25	12.84	

Note: High accuracy of in-house ELISpot assay in methodology

PBMCs from 5 CHB patients were repeatably examined for three times under the same conditions by the same operator. Additionally, the PBMCs from indicated CHB patient were divided into 3 equal parts and conducted by 3 independent operators respectively. As shown in Table S4, the intra-assay CV and inter-assay CV were 7.26% and 7.95%, respectively. Representative spot plots were presented in Fig. S10.

Supplementary Figures

Figure S1:

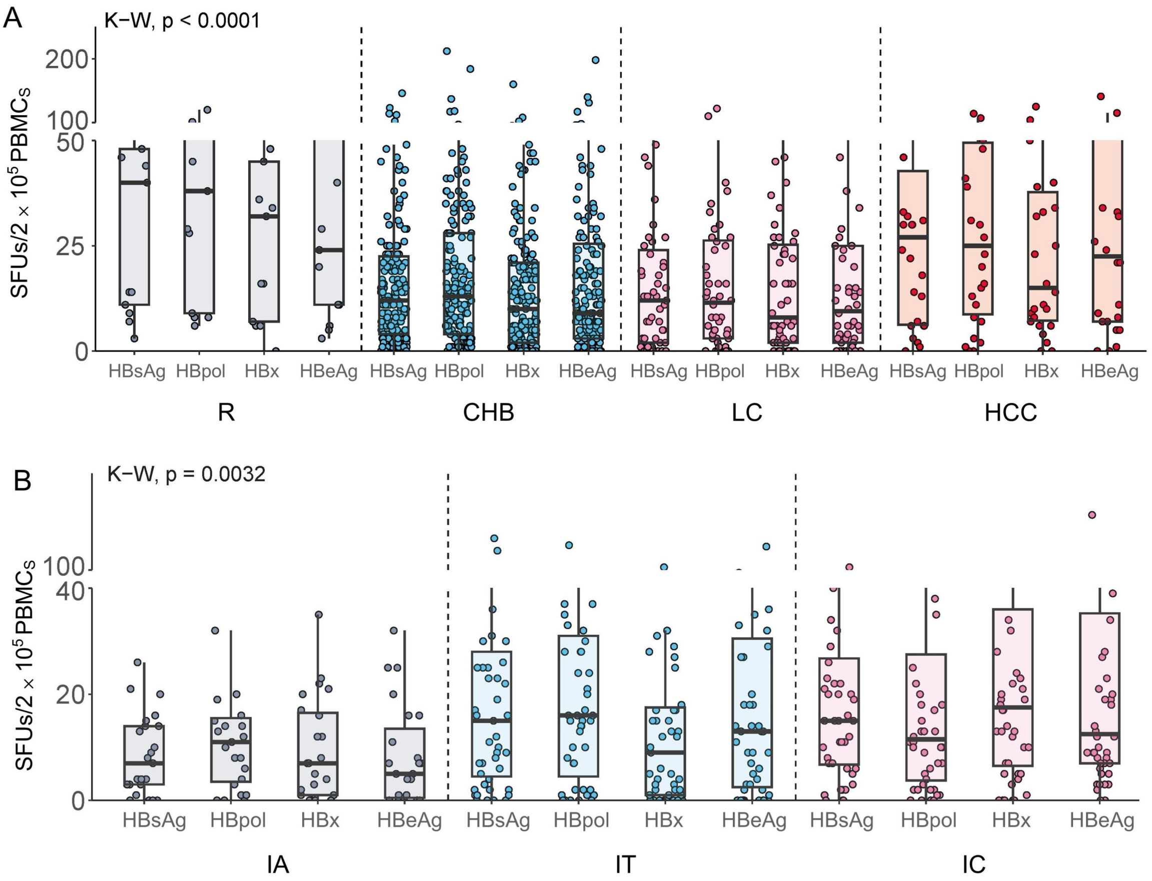

Fig. S1 Specific T cells reactive to different HBV proteins in different disease phases of HBV-infected patients. (A) Specific T cells (SFUs) reactive to each HBV antigen (HBsAg, HBpol, HBx, HBeAg) in R, CHB, LC and HCC patients. R, acute resolved patients (n = 13); CHB, chronic hepatitis B (n = 203); LC, liver cirrhosis (n = 52); HCC, hepatocellular carcinoma (n = 26). **(B)** Specific T cells (SFUs) reactive to each HBV antigen (HBsAg, HBpol, HBx, HBeAg) in IA, IT, and IC phases of CHB patients. IA, immune active phase (n = 23); IT, immune tolerant phase (n = 44); IC, immune inactive carrier phase (n = 47). The medians (interquartile range) are presented and statistical analyses were performed using Mann-Whitney test (M-W) between two groups and Kruskal-Wallis test (K-W) across more than two groups.

Figure S2:

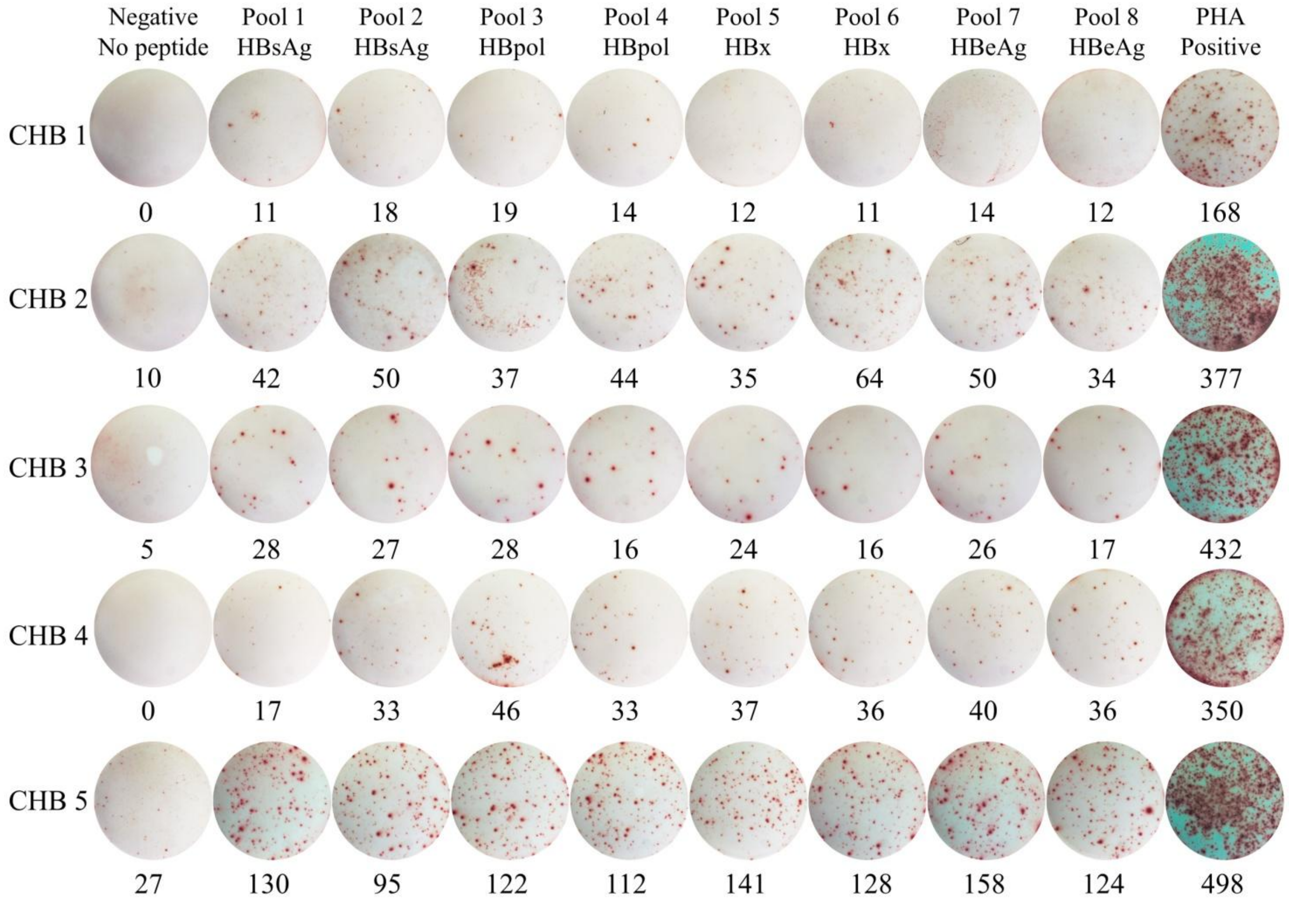

Fig. S2 Dot plots of IFN-γ SFUs from five representative CHB subjects in the in-house ELISpot assay. PBMCs from each patient were seed into 10 wells and co-cultured for 20 hours with no peptide, eight peptide pools and PHA, respectively. Then IFN-γ was detected by ELISpot assay. PHA: Phytohemagglutinin.

Figure S3:

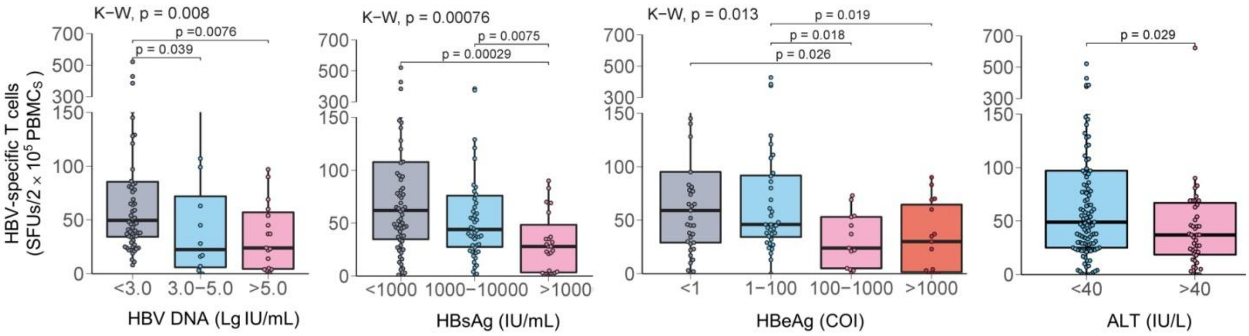

Fig. S3 HBV-specific T cell reactivity in CHB patients undergoing NUCs monotherapy. A cohort of CHB patients undergoing NUCs monotherapy (n = 167) were grouped by HBV viral load (<3.0, n = 62; 3.0-5.0, n = 12; >5.0, n = 19), HBsAg level (<1000, n = 66; 1000-10000, n = 50; >10000, n = 27), HBeAg level (<1, n = 37; 1-100, n = 36; 100-1000, n = 13; >1000, n = 15) and ALT level (<40, n = 117; >40, n = 47). Then reactive HBV-specific T cells (SFUs) were compared across the groups. The medians (interquartile range) are presented and statistical analyses were performed using Mann-Whitney test (M-W) between two groups and Kruskal-Wallis test (K-W) across more than two groups.

Figure S4:

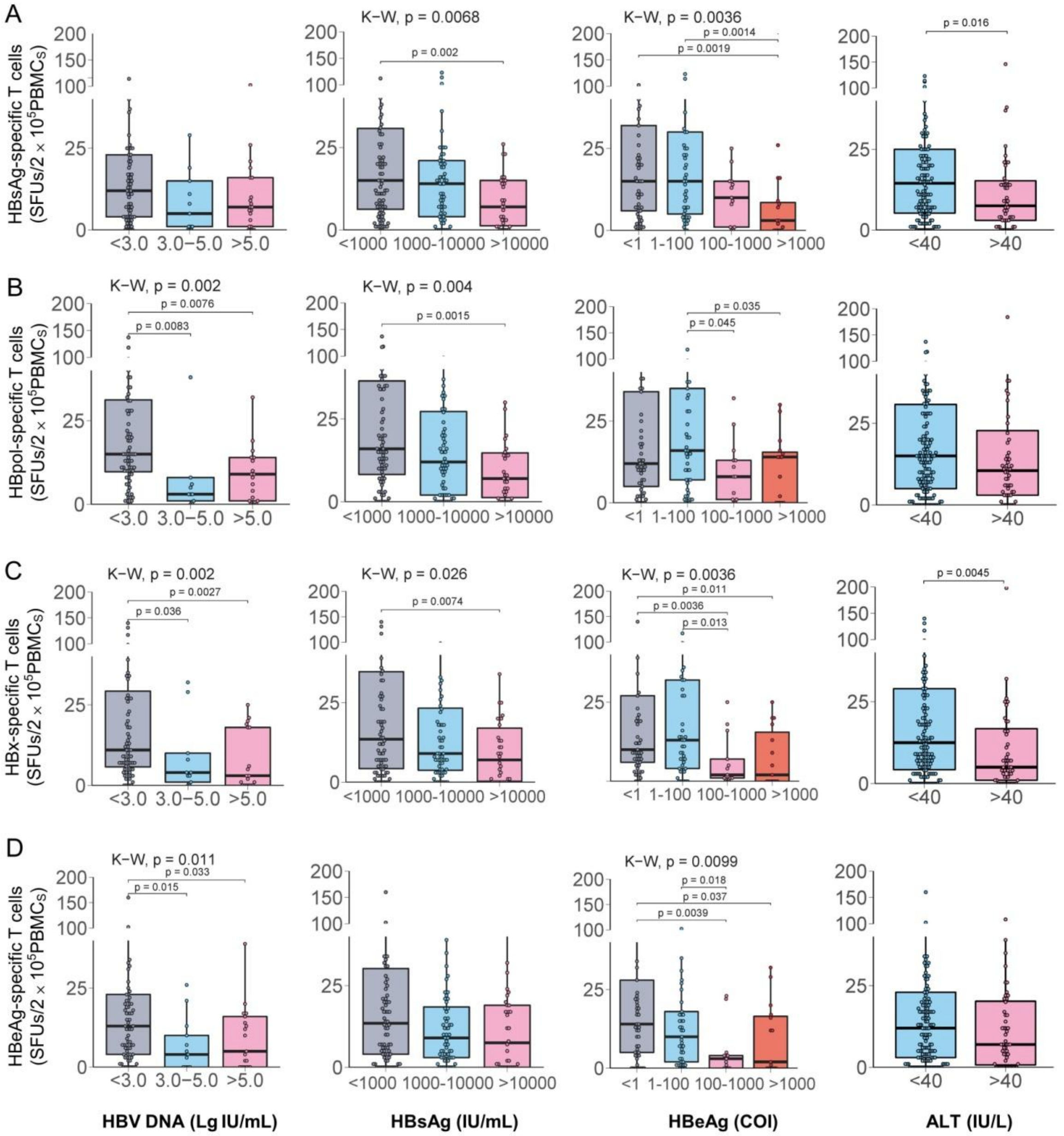

Fig. S4 Stratified analyses of specific T cells reactive to each HBV protein in CHB patients with different sero-virological profiles. According to the SFUs reactive to each peptide pool in the *ex vivo* ELISpot assay, HBsAg-specific T cells **(A)**, HBpol-specific T cells **(B)**, HBx-specific T cells **(C)**, and HBeAg-specific T cells **(D)** were further calculated and stratified analyzed in CHB patients grouped by HBV DNA load (<3.0, n=62; 3.0-5.0, n=12; >5.0, n=19), HBsAg level (<1000, n=66; 1000-10000, n=50; >10000, n=27), HBeAg level (<1, n=37; 1-100, n=36; 100-1000, n=13; >1000, n=15) or ALT level (<40, n=117; >40, n=47).

Figure S5:

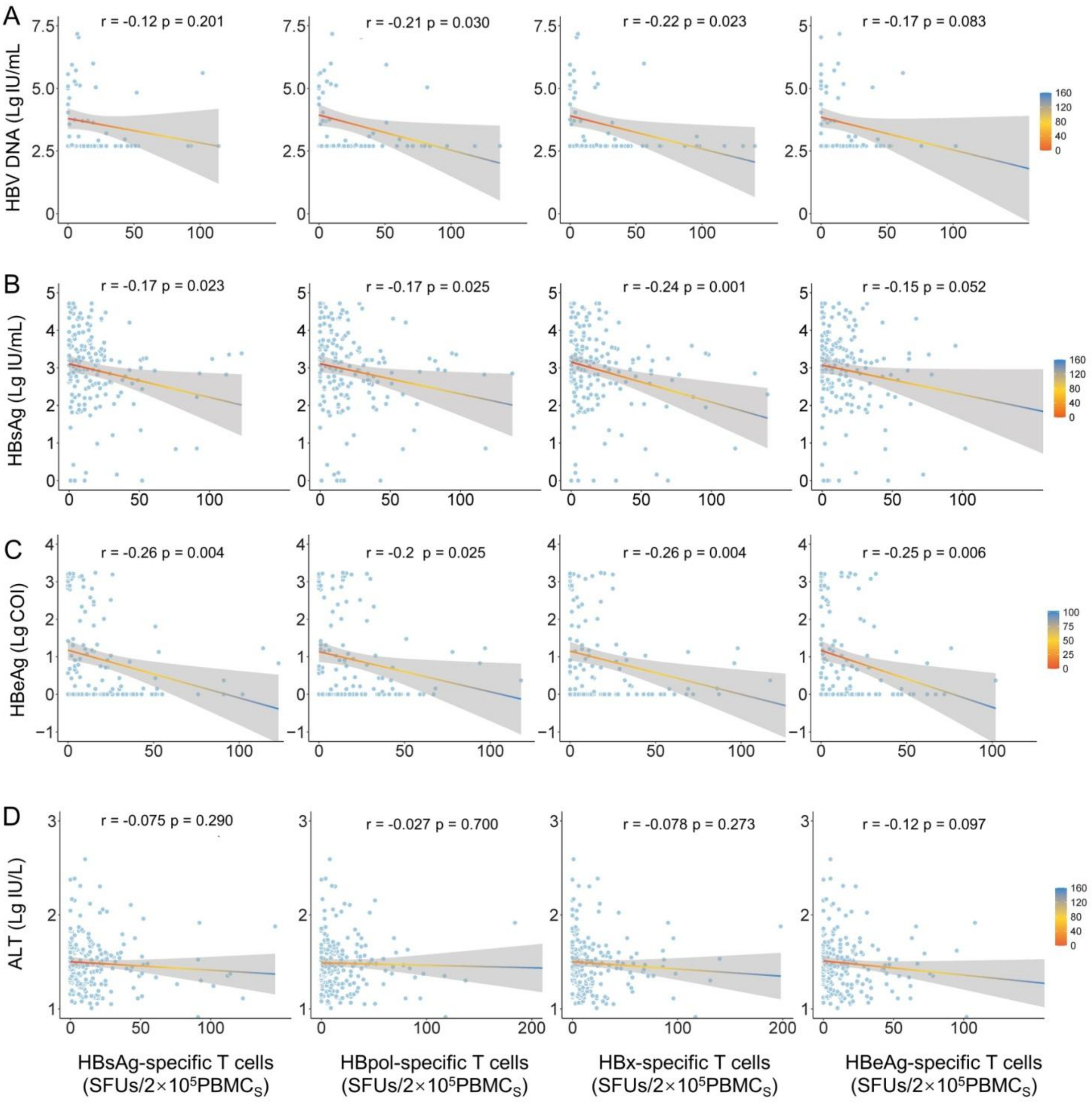

Fig. S5 Correlation between the specific T cells reactive to each HBV protein and sero-virological parameters in CHB patients. Spearman correlation tests between HBsAg-, HBpol-, HBx-, or HBeAg-specific T cells and HBV DNA load (**A**, n = 110), HBsAg level (**B**, n = 180), HBeAg level (C, n = 126), or ALT level (**D**, n = 202) were performed.

Figure S6:

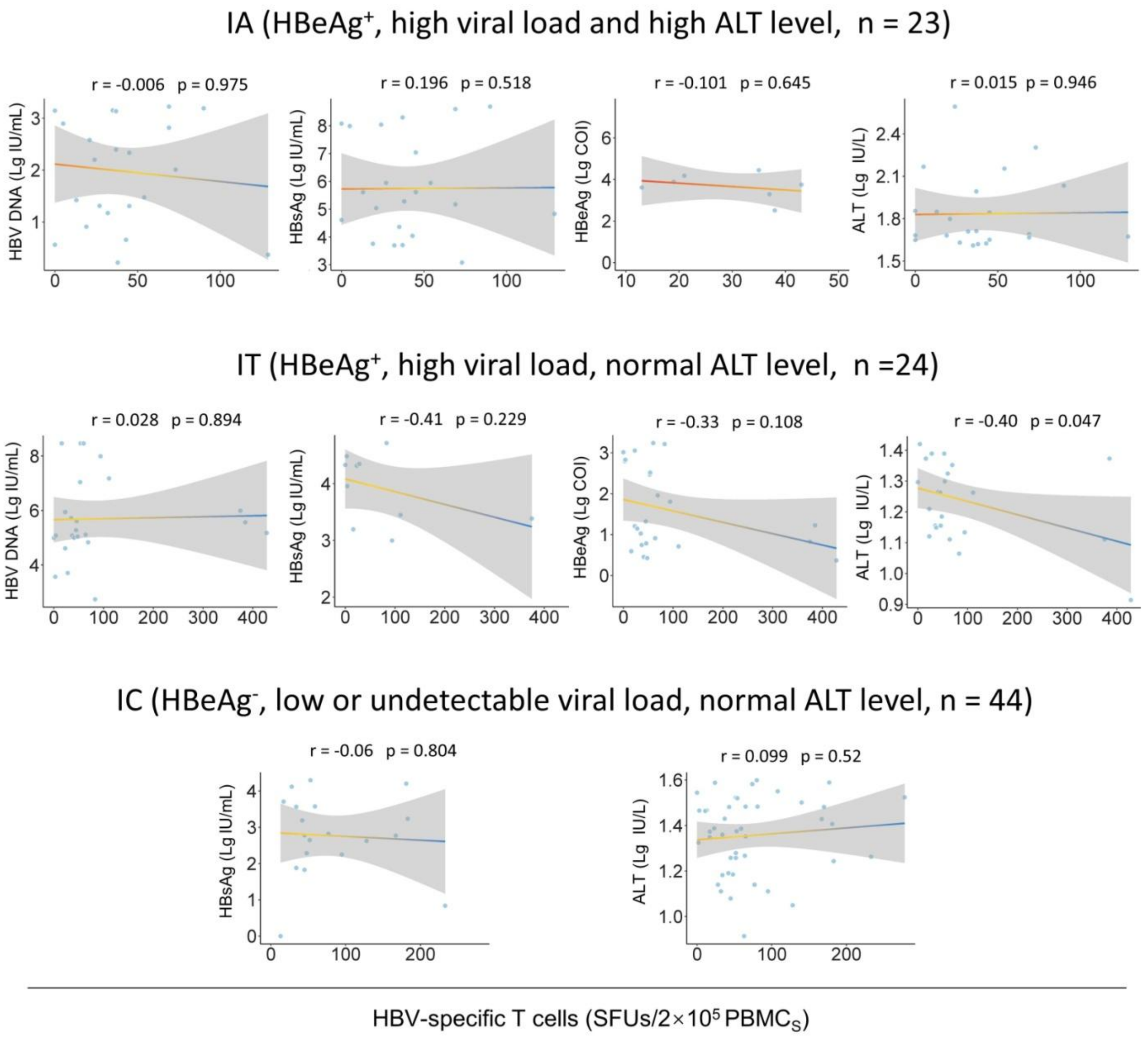

Fig. S6 Correlation between HBV-specific T cells reactivity and sero-virological parameters in CHB patients at IA, IT or IC phase. In IA, IT or IC group, Spearman correlation tests between HBV-specific T cells (SFUs) and HBV DNA load, HBsAg level, HBeAg level, or ALT level were performed.

Figure S7:

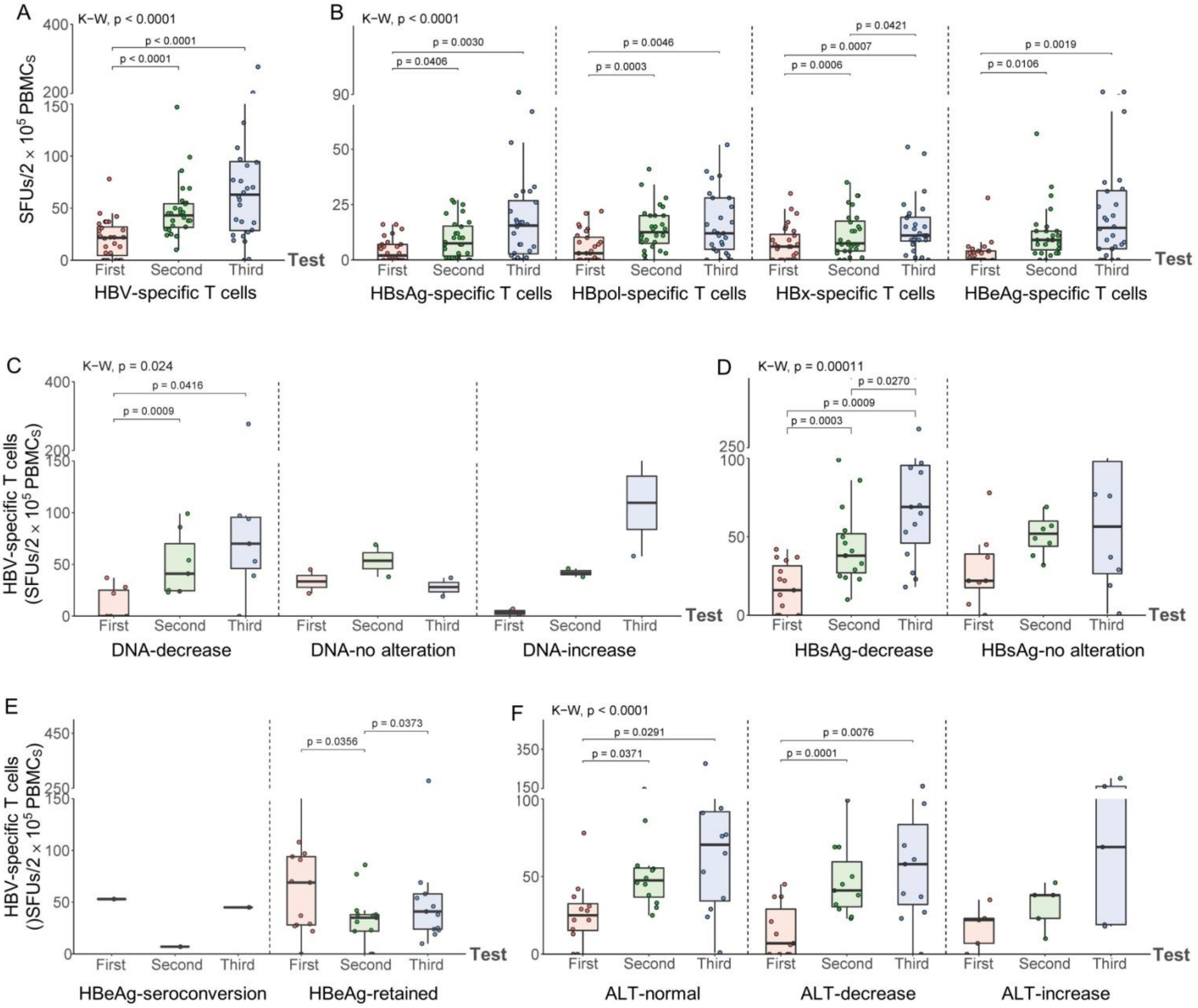

Fig. S7 Dynamic reactivity of HBV-specific T cells during different sero-virological courses in 28 CHB patients undergoing NUCs monotherapy. 28 CHB patients undergoing NUCs monotherapy were followed by HBV-specific T cell detection and sero-virological parameters collections for three times at an interval of 3-5 months. Dynamic changes of total HBV-specific T cells **(A)** and specific T cells reactive to each HBV protein **(B)** were presented. Then, the dynamic changes of HBV-specific T cells in CHB patients with different fluctuation courses of HBV DNA load (decrease, n = 7; no alternation, n = 2; increase, n = 2)**(C)**, HBsAg level (decrease, n = 15; no alternation, n = 7)**(D)**, HBeAg seroconversion (HBeAg-seroconversion, n = 1) or kept positive (HBeAg-retained, n =

12)**(E)**, or ALT level (normal, n = 12; decrease, n = 11; increase, n = 5)**(F)** were shown. The patients who achieved DNA fluctuations (increase or decrease) >30% were defined as the DNA-increase or DNA-decrease group, and the other patients were defined as DNA-no alternations group. HBsAg-decrease was defined as an amplitude decrement of more than 30%. CHB patients who experienced a positive HBeAg serology (HBeAg COI >1) at first and seroconverted (HBeAg COI <1) later during the follow-up period were defined as the HBeAg-seroconversion group. ALT-decrease was defined as a decline to the normal range (<40 IU/L) or changed more than 30%, while ALT that rose more than 30% or beyond 40 IU/L was defined as ALT-increase. Medians (interquartile range) are presented and statistical analyses were performed using the paired, two-tailed Student's t-test between two groups and Kruskal-Wallis test (K-W) across more than two groups.

Figure S8:

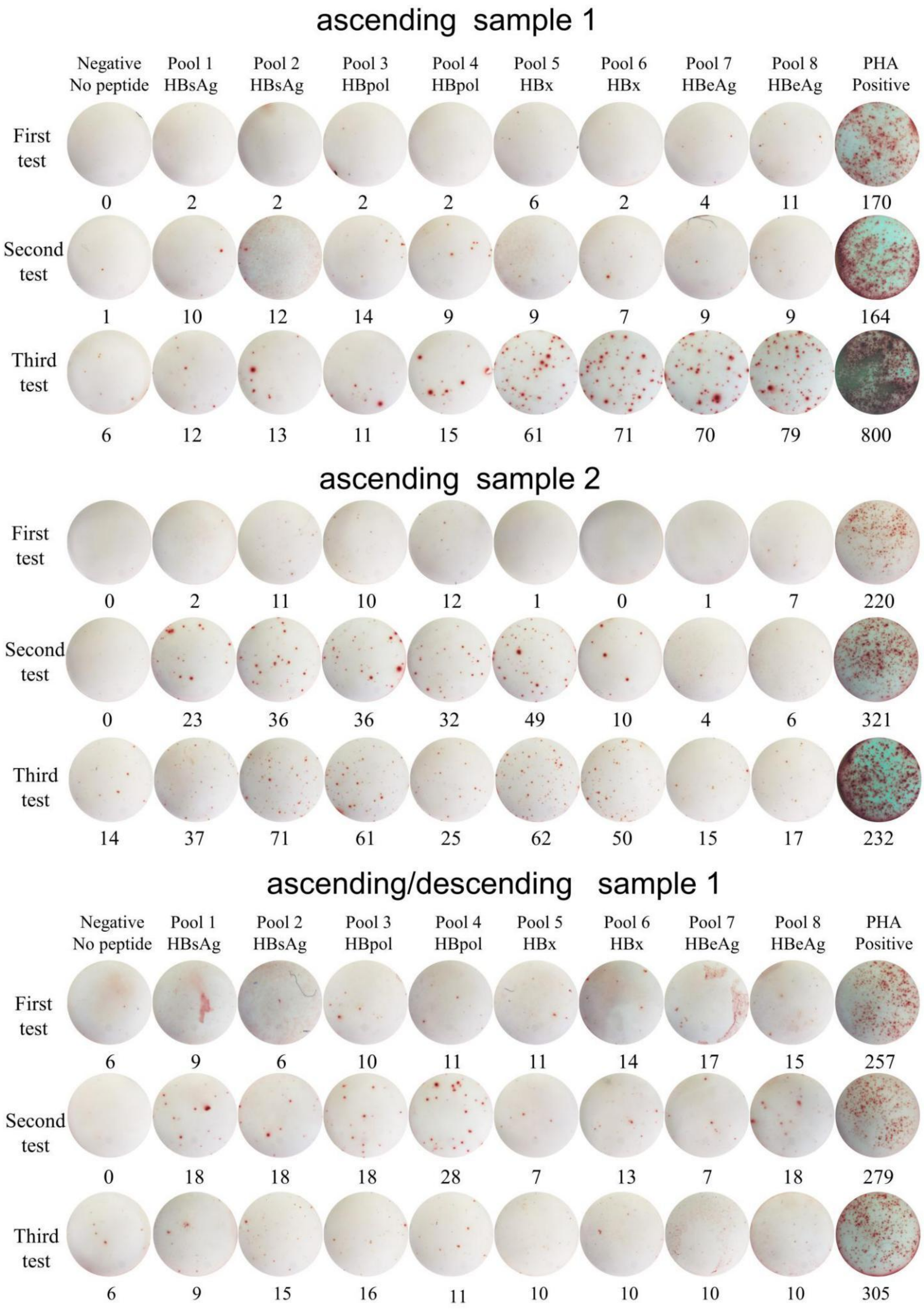

ascending sample 1
Negative No peptide
Pool 1 HBsAg
Pool 2 HBsAg
Pool 3 HBpol
Pool 4 HBpol
Pool 5 HBx
Pool 6 HBx
Pool 7 HBeAg
Pool 8 HBeAg
PHA Positive
First test
0 2 2 2 2 6 2 4 11 170
Second test
1 10 12 14 9 9 7 9 9 164
Third test
6 12 13 11 15 61 71 70 79 800
ascending sample 2
First test
0 2 11 10 12 1 0 1 7 220
Second test
0 23 36 36 32 49 10 4 6 321
Third test
14 37 71 61 25 62 50 15 17 232
ascending/descending sample 1
Negative No peptide
Pool 1 HBsAg
Pool 2 HBsAg
Pool 3 HBpol
Pool 4 HBpol
Pool 5 HBx
Pool 6 HBx
Pool 7 HBeAg
Pool 8 HBeAg
PHA Positive
First test
6 9 6 10 11 11 14 17 15 257
Second test
0 18 18 18 28 7 13 7 18 279
Third test
6 9 15 16 11 10 10 10 10 305

Fig. S8 Dot plots of IFN-γ ELISpot from the representative CHB patients undergoing ascending or ascending/descending courses of HBV-specific T cells. 33 CHB patients were followed by HBV-specific T cell detection and sero-virological parameters collections for three times at an interval of 3-5 months. According to the dynamic courses of HBV-specific T cells (SFUs), patients were categorized as ascending (n =22), ascending/descending (n=7), stationary (n=2), descending (n=1), and descending/ascending (n = 1) groups. The SFUs of HBV-specific T cells which increased or decreased more than 50% than last test was defined as ascending or descending during the follow-up period.

Figure S9:

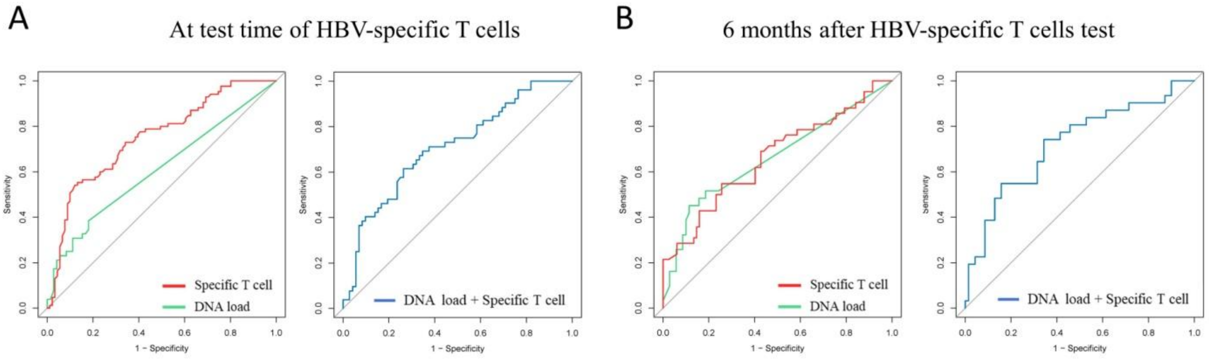

	Marker	Patient number	Normal/ Abnormal	AUC	95% CI	Cut-off level	Specificity (%)	Sensitivity (%)	PPV (%)	NPV (%)	P¹	P²
At test time	DNA load	124	72/52	0.609	0.528-0.691	540	38.5%	81.9%	60.6%	64.8%	0.008	0.03
	Specific T cell	176	91/85	0.750	0.678-0.822	89	55.3%	86.8%	79.7%	67.5%	<0.001	0.10
	DNA load + Specific T cell	124	72/52	0.709	0.617-0.802	0.48	73.6%	61.5%	72.6%	62.7%	<0.001	
6 months after test	DNA load	101	70/31	0.661	0.553-0.769	3500	88.6%	45.2%	78.5%	63.5%	0.003	0.45
	Specific T cell	124	82/42	0.662	0.557-0.768	81	74.4%	54.8%	76.2%	52.3%	0.002	0.59
	DNA load + Specific T cell	101	70/31	0.722	0.610-0.835	0.33	74.2%	65.7%	85.2%	48.9%	0.0001	

Fig. S9 Predictive power of cross-sectional reactivity of HBV-specific T cells for liver hepatitis progression in CHB patients. 176 CHB patients beyond the 200 CHB patients in Fig. 6 were divided into normal (ALT <40 IU/L) group and abnormal (ALT >40 IU/L) group of liver function at the test time of HBV-specific T cells or 6 months after the test. ROC curve analyses of DNA load (IU/mL), HBV-specific T cells (SFUs/4×10^5 PBMCs), and a combination were performed to predict liver hepatitis progression at the test time of HBV-specific T cells **(A)** and 6 months after HBV-specific T cells test **(B)**, using R package pROC, and summarized in table. ROC, receiver operating characteristic; AUC, area under the curve; PPV, positive predictive value; NPV, negative predictive value. The p^1 values represent the significance of model. The p^2 values represent the significance of difference between the AUC of combined markers (DNA load + Specific T cell) and single predictor.

Figure S10:

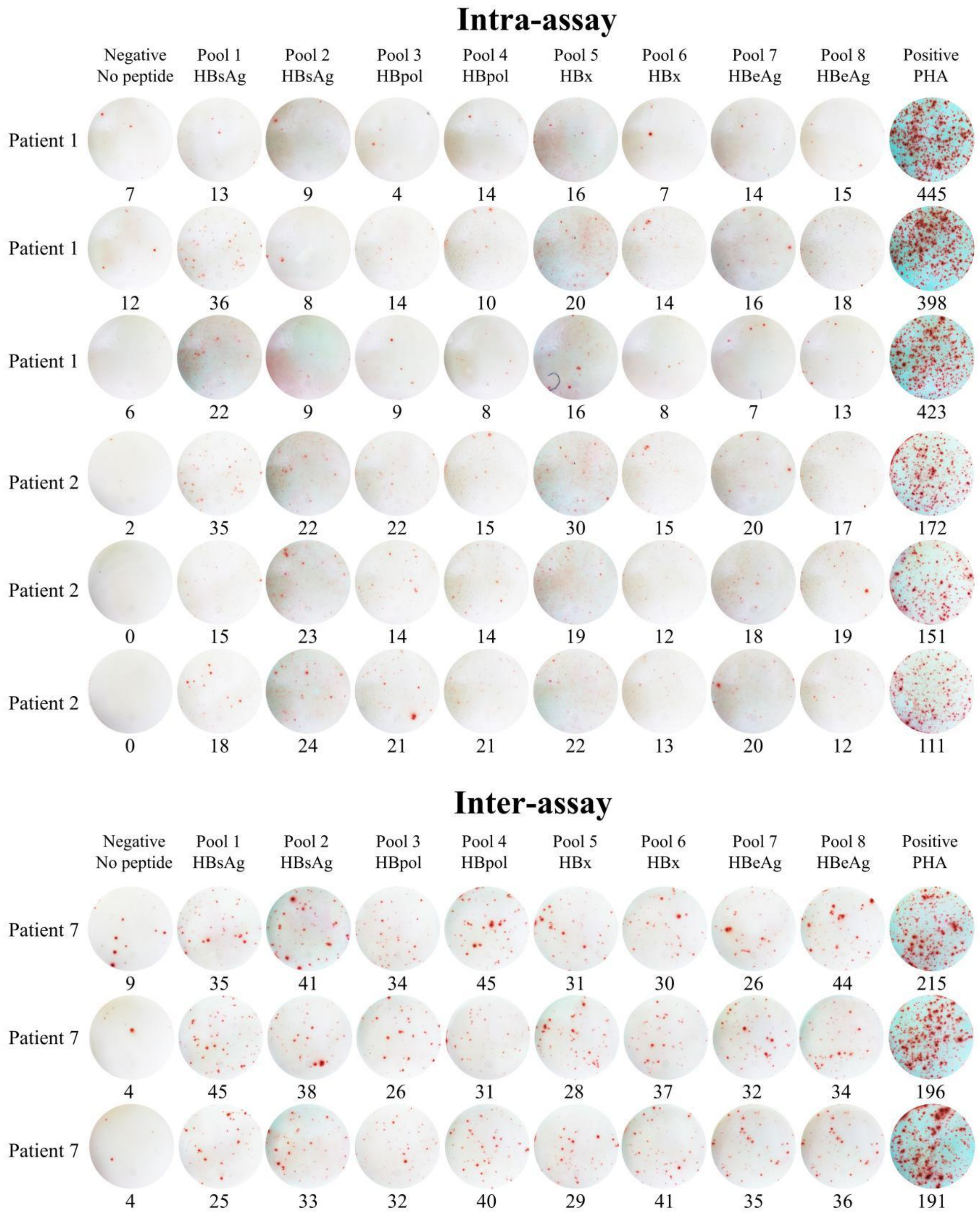

Fig. S10 High reproducibility of the in-house ELISpot assay. For intra-assay, PBMCs from each CHB patient (n = 5) were detected three times under the same conditions by a single operator. For inter-assay, PBMCs from each CHB patient (n = 3) were divided into three equal parts and detected by three independent operators respectively. Dot plots of IFN-γ ELISpot from representative subjects were presented.
